# Genomic Monitoring of a Reintroduced Butterfly Uncovers Contrasting Founder Lineage Survival

**DOI:** 10.1111/eva.70074

**Published:** 2025-02-06

**Authors:** Georgina Halford, Dirk Maes, Carl J. Yung, Sam Whiteford, Nigel A. D. Bourn, Caroline R. Bulman, Philippe Goffart, Jenny A. Hodgson, Ilik J. Saccheri

**Affiliations:** ^1^ Department of Evolution, Ecology and Behaviour University of Liverpool Liverpool UK; ^2^ Research Institute for Nature and Forest (INBO) Brussels Belgium; ^3^ Radboud Institute for Biological and Environmental Sciences (RIBES) Radboud University Nijmegen The Netherlands; ^4^ Butterfly Conservation Wareham, Dorset UK; ^5^ Département d'Étude du Milieu Naturel et Agricole/Service Public de Wallonie (DEMNA/SPW) Gembloux Belgium

**Keywords:** admixture, Chequered skipper, conservation translocation, genetic bottleneck, *Lepidoptera*, population genomics, whole genome sequencing, *Wolbachia*

## Abstract

Genetic factors can have a major influence on both short‐ and long‐term success of reintroductions. Genomic monitoring can give a range of insights into the early life of a reintroduced population and ultimately can help to avoid wasting limited conservation resources. In this study, we characterise the genetic diversity of a reintroduced *Carterocephalus palaemon* (Chequered Skipper butterfly) population in England with respect to the spatial genetic structure and diversity of the source populations in south Belgium. We aim to evaluate the success of the reintroduction, including the effectiveness of the donor sampling strategy, and assess genetic vulnerabilities that may affect the population's future. We also use an isolation‐by‐distance approach to make quantitative inferences about dispersal, and we explore covariance between host mitochondrial and *Wolbachia* genomes. We find that, four generations following the initial release, the reintroduced population, founded by 66 wild‐caught adults, has an effective size of c. 33, yet has retained similar levels of genomic heterozygosity to those in the source subpopulations in Belgium and shows low levels of inbreeding. However, the restricted number of founders and variance in reproductive success among the surviving families have resulted in a higher level of kinship, likely to result in somewhat higher rates of inbreeding in the future. Furthermore, there is a distinct split between two source landscapes in Belgium, and all genomic evidence suggests that the reintroduced population is descended from only one of these landscapes (called Fagne). We discuss potential causes behind these results, including whether *Wolbachia* strains are causing genetic incompatibility between clades. We conclude that a conservative strategy for any further translocations would prefer Fagne sites as sources because of the strong evidence of their ability to survive. However, our results warrant further investigation into the reasons for the divergence found in Belgium.

## 1 Introduction

Habitat loss and fragmentation are among the primary causes of declines in biodiversity (Rands et al. 2010). Increased amounts of habitat destruction have led to the division of habitat into smaller and isolated fragments, leading to widespread species declines and extinctions (Haddad et al. 2015). Reintroductions, defined as ‘the intentional movement and release of an organism inside its indigenous range from which it has disappeared’ (IUCN/SSC 2013), of individuals from demographically stable source populations to suitable unoccupied habitat can be applied as a conservation measure to reverse local extinctions (Taylor et al. 2017). One of the most famous invertebrate reintroduction success stories is that of the Large Blue butterfly (*Phengaris arion*), which went regionally extinct from the British Isles in 1979. Starting with a Swedish donor population in 1983, a series of reintroductions have resulted in a self‐sustaining meta‐population in southwest England (Thomas, Simcox, and Clarke 2009).

Over recent decades, there has been increased awareness of the role that genetic factors have in the success of reintroductions (Armstrong and Seddon 2008; Davis et al. 2021; Weeks et al. 2011; Zavodna et al. 2015). A key objective for practitioners is to maximise genetic diversity in order both to promote adaptive potential under environmental stresses and to avoid fitness reductions via inbreeding depression (Andersen et al. 2014; Zavodna et al. 2015). Avoidance of reduced genetic diversity relies upon: (i) the genetic composition of the founder individuals, which includes the number of individuals released as well as their relatedness; (ii) the differential survival of the founding lineages; and (iii) the population growth rate in the generations following reintroduction (Frankham, Ballou, and Briscoe 2002; La Haye et al. 2017).

In many cases, low population size and isolation associated with reintroductions can increase the risk of inbreeding depression (Mueller et al. 2022). Inbreeding depression typically affects core physiological traits that are strongly linked to fertility, fecundity, and viability, meaning that it can have immediate effects on reproductive output (Keller and Waller 2002). In addition to the genetic composition of individuals, broader species‐level properties can affect how susceptible a reintroduced population is to inbreeding depression. For example, in butterflies, it has been shown that mating behaviour, landscape structure and dispersal ability can all affect how susceptible a particular species is (Nonaka et al. 2019). Moreover, inbreeding depression is expected to be much more common in small habitat patches or networks, such as those that follow a reintroduction, elevating the risk of failure through extinction in reintroduced populations (Andersen et al. 2014).

Due to the potential effects of inbreeding and low genetic diversity, many reintroduction projects aim for a sampling approach that maximises genetic diversity by using several source sites, assumed or known to have genetically diverged populations (Porter and Ellis 2011; Zavodna et al. 2015; Parmesan et al. 2023; Todd et al. 2023). However, when taken to extremes, this approach runs the risk of outbreeding depression, which can result from phenotypes that are poorly adapted to the local conditions of the release site or genetic incompatibilities between released individuals (Byrne and Silla 2020).

Given the risks and benefits involved (Weeks et al. 2011), there are thus two major rationales for using population genetic information in reintroduction projects. Firstly, in source sampling strategies, practitioners should consider the genetic structure of the source populations, in the context of genetic drift, gene flow and local adaptation, when deciding whether to reintroduce individuals from one or more sources (Todd et al. 2023). Secondly, the reintroduced population's genetics should be monitored to assess (and mitigate) rates of inbreeding and patterns of lineage survival (Mueller et al. 2022).

In the case of insects, an issue for genetic sampling comes from the small size of the organisms, particularly when using methods such as whole genome sequencing, which ideally require > 100 ng of DNA to make a genomic library. Usually, the sampling method used results in the death of the insect (Richards and Murali 2015), which is highly problematic for rare insects or small populations. Therefore, a step‐change in reintroduction biology is possible when tissue sampling methods that have a negligible effect on survival are developed (Gershman et al. 2021). In butterflies, wing‐clips have been used as a non‐destructive method of obtaining DNA. However, many of these studies have been limited to mitochondrial (Lushai et al. 2000) or microsatellite assays (Roland, Keyghobadi, and Fownes 2000; Saarinen, Austin, and Daniels 2010; Andersen et al. 2014; Vanden Broeck et al. 2017; Davis et al. 2021; De Ro et al. 2021). Here, we show that a practical and minimally invasive method of wing clipping can be used to obtain enough DNA for individual whole genome sequencing.

As compared to microsatellite or mitochondrial assays, whole‐genome single nucleotide polymorphisms (SNPs) carry distinct advantages for estimating genome‐wide diversity and reconstructing demography (Väli et al. 2008), as well as offering the potential to estimate genetic load (Bertorelle et al. 2022). Whole genome sequencing methods provide a high‐resolution view of the genome, providing more detailed insights into the long‐term viability and extent of inbreeding in reintroduced populations (Grossen et al. 2018). Moreover, as whole genome sequencing necessarily sequences all the genomes in the sample, we obtain data not only on the nuclear genome but also the mitochondrial genome, and any cobiont infections (bacteria, fungi, viruses) that may affect the success of a reintroduction programme. In particular, the endosymbiotic bacterium *Wolbachia*, which is widespread among insects, has been flagged as being of potential conservation concern for several insect species (Bordenstein and Werren 2007; Dincă et al. 2018; Hamm et al. 2014; Zhu and Liu 2024).

In 2018, the charity Butterfly Conservation began a reintroduction of *Carterocephalus palaemon* (Chequered Skipper) into Fineshade Wood in the Rockingham Forest landscape in England's East Midlands (henceforth “Fineshade”). *Carterocephalus palaemon* was always considered rare in England, being confined to a band of woods and associated limestone grasslands in this region, and became extinct here in 1976, likely through changes in habitat management, land use changes, neglect of its habitat, and a very dry summer (Moore 2004). Within the British Isles, the species is now confined to a small area of western Scotland (Bourn et al. 2024). Adult butterflies for reintroduction were taken from six different sites across the Fagne‐Famenne‐Calestienne region in Belgium (Maes et al. 2019), with the hope that they represented an appropriate ecotype and sufficient genetic diversity. Following translocations into Fineshade, the reintroduced population of *C. palaemon* has been monitored through survey methods including transects and timed counts (Bourn et al. 2024; Wildman et al. 2024). So far, the population has increased in area occupied, spreading further from the release location, providing positive indications of a self‐sustaining population. The reintroduction programme aims to establish functioning meta‐populations in the Rockingham Forest landscape, with the species moving across the landscape and colonising sites naturally (Hanski 1998). Previous research suggested that *C. palaemon* , while colonial, with large numbers of individuals moving less than 100 m, was also occasionally seen moving away from breeding areas and even possibly a few kilometres (Ravenscroft 1992). Thus, planning by Butterfly Conservation has assessed five smaller ‘sub‐landscapes’ with potential for natural colonisation internally (sites mostly within 500 m) but separated from each other by more than 1.5 km of intensive farmland (Bourn et al. 2024). Recently, further translocations have taken place into a site within a different sub‐landscape, with the aim that two meta‐populations will be established (Bourn et al. 2024).

While the initial success of the reintroduction has been established, population size estimates on their own provide limited information for assessing the potential for long‐term persistence or expansion of a newly established population. We also need to consider genetic attributes of the reintroduced population and the species' capacity to colonise neighbouring suitable habitat (Armstrong and Seddon 2008). Considerable work has been conducted on *C. palaemon* ecology, in particular with respect to habitat requirements (Ravenscroft 1994; Moore 2004; Halford et al. 2024; Wildman 2023), but there is little concrete information about its dispersal across longer distances, an important piece of missing information in light of the aims to create a meta‐population. In this study, we characterise the genetic diversity of the reintroduced *C. palaemon* population with respect to the spatial genetic structure and diversity of the source populations, aiming to evaluate the success of the reintroduction, including the effectiveness of the donor sampling strategy. We also use an isolation‐by‐distance approach to make quantitative inferences about dispersal and explore covariance between host mitochondrial and *Wolbachia* genomes.

We applied whole genome sequencing of wing‐clips sampled from the reintroduced population in Fineshade and the source Fagne‐Famenne‐Calestienne region in Belgium to answer the following questions: (i) was the reintroduction donor sampling strategy adequate for representing the source region's genetic diversity; (ii) which, if any, of the source populations were most successful in survival and breeding after reintroduction (can we see evidence of descendants from a few or all source populations); (iii) what is the dispersal distance of *C. palaemon* ; (iv) what was the effective size of the reintroduction bottleneck, and how much genetic diversity was retained; (v) Is the level of inbreeding in the reintroduced population a concern; and (vi) is *Wolbachia* infection associated with differences in reproductive success between founder lineages?

## 2 Materials and Methods

### 2.1 Reintroduction of *Carterocephalus palaemon* to England

The reintroduction of *C. palaemon* into England began in 2018. The Rockingham Forest landscape, and in particular Fineshade Wood, was chosen as the initial reintroduction site after qualitative assessments, including the presence of larval host plants, vegetation structure and extent of open space to determine which patches would be suitable reintroduction locations (Bourn et al. 2024). Furthermore, Fineshade Wood was the last stronghold of the species in England until extinction in 1976. Source populations were selected based on species distribution models combining distribution data and environmental variables covering several potential source regions in Belgium, The Netherlands, and Scotland. Based on the outputs of these models and additional expert knowledge, the Fagne‐Famenne‐Calestienne region of Wallonia, south Belgium, was chosen (Maes et al. 2019). An initial translocation of 42 adults (32 females and 10 males) from Belgium to Fineshade Wood took place in 2018. A second release of 24 adults (12 females and 12 males) occurred in 2019 (Wildman 2023; Bourn et al. 2024). Individuals were collected from 6 sites spanning the Fagne‐Famenne‐Calestienne region in order to maximise the initial genetic diversity of the reintroduced individuals. Further translocations have also taken place since the collection of genetic material for this study (5 additional females were released in 2022 into Fineshade Woods). *Carterocephalus palaemon* is a univoltine species, overwintering at the larval stage.

### 2.2 Wing‐Clip Sample Collection

**FIGURE 1:**
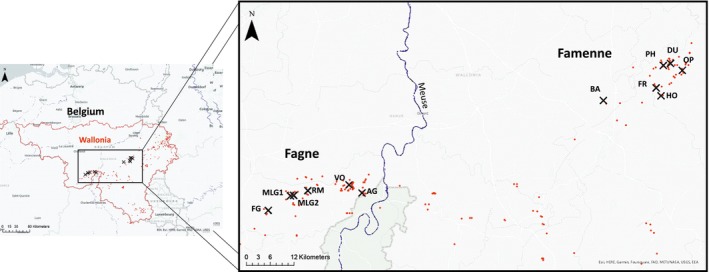
Map of the reintroduction source region in Belgium. The 12 subpopulations sampled for wing‐clips are shown (black crosses), which include the subpopulations used as sources for the reintroduction: Fagnolle (FG), Matagne‐la‐Grande 1 & 2 (MG1 & MG2), Romerée (RM), Vodelée (VO), Agimont (AG), Baillonville (BA), Hotton (HO), Fronville (FR), Petit‐Han (PH), Durbuy (DU) and Oppagne (OP). These sites are located in two labelled landscapes: Fagne and Famenne, on opposite sides of the river Meuse. Red dots indicate any *C. palaemon* occurrences in Wallonia (the Southern part of Belgium, outlined in red) recorded between 2010 and 2019 (provided by the Public Service of Wallonia).

Tissue samples were taken from a total of 12 sites across the Fagne‐Famenne‐Calestienne region in Belgium between 1 and 12 June 2021 (Figure 1). These sites (or local subpopulations) were chosen to include the six sites used as sources for the reintroduction and to represent the meta‐population in which they are embedded, based on observed *C. palaemon* occurrences, accessibility, and distance from other chosen locations. The sites fall into two clusters, the Fagne landscape (west of the river Meuse) and the Famenne landscape (east of the river Meuse) (Figure 1). It was not possible to sample the intervening areas of land due to either small populations or occurrence on protected sites. Adult butterflies were non‐destructively sampled in the field by removing a very small piece of wing (~2 mm^2^), taken from the back edge of the hind wing using tweezers. Wing‐clips were immediately placed in 100% ethanol. This non‐invasive sampling approach resembles normal wear and tear of the butterfly. All sample locations were recorded using a GPS. A total of 128 clips (94 males and 34 females) were taken (Table 1). Of these, we began library preparation with 112, with 16 being excluded due to changes in genomic library preparation methods. Wing‐clips were also taken using the same method from individuals at the reintroduction site in Fineshade. A total of 15 wing‐clips were taken, 2 in June 2021 (due to weather conditions limiting sampling) and 13 in late May–June 2022, from adults located across the occupied site but mostly within 2 km of each other (Figure S1). We began library preparation with a total of 127 individuals from Belgium and Fineshade combined.

**TABLE 1:** Number of *C. palaemon* wing‐clip samples collected for DNA sequencing from each site in Belgium (the source region), and Fineshade (the reintroduction site).

Site	Wing‐clips collected (male, female)	Landscape
Fagnolle	12 (12, 0)	Fagne
**Matagne‐la‐Grande1**	12 (8, 4)	Fagne
**Matagne‐la‐Grande2**	13 (13, 0)	Fagne
Romerée	13 (10, 3)	Fagne
**Vodelée**	13 (12, 1)	Fagne
Agimont	1 (1, 0)	Fagne
Baillonville	2 (1, 1)	Famenne
Hotton	13 (4, 9)	Famenne
**Fronville**	12 (5, 7)	Famenne
Petit Han	12 (11, 1)	Famenne
**Durbuy**	12 (8, 4)	Famenne
**Oppagne**	13 (9, 4)	Famenne
Fineshade	15 (10, 5)	Fineshade

*Note:* Male/female numbers are shown in brackets. Sites that were also used as sources for the reintroduction are highlighted in bold. All samples in Belgium were collected in 2021, whereas collection in Fineshade took place in 2021 and 2022.

### 2.3 DNA Extraction and Whole Genome Sequencing

*Carterocephalus palaemon* has a haploid genome size of 394.5 Mb and 31 chromosomes (Lohse et al. 2023). To sequence our samples, we used a whole genome rather than a reduced‐representation library approach. All sequences were individually barcoded and mapped to a Darwin Tree of Life (DToL) *C. palaemon* reference sequence (GCA_944567795.1 (Lohse et al. 2023)) for analysis. Barcoded adaptors were obtained from the Centre for Genomic Research (CGR), University of Liverpool. Steps are outlined below.

#### 2.3.1 Sample Storage and DNA Extraction

Field‐collected samples were stored in ethanol and in a fridge until used. Ethanol was removed from the wing‐clips, and clips were left to dry overnight in a 30°C incubator/oven. DNA was extracted from the wing‐clips by adding 30 μL of TE (Qiagen) to the wing‐clip in a 1.5 mL Eppendorf microfuge tube. The wing‐clips were then homogenised using a micro pestle (Starlab I1415‐5390) to maximise DNA yield. The DNA was then quantified using the Quant‐IT dsDNA Assay high sensitivity kit and a Qubit fluorometer. Six samples were excluded at this stage due to insufficient DNA, including 1 sample from Fineshade, leaving 121 samples that were further processed and sequenced.

#### 2.3.2 Library Preparation

Each of the following steps was conducted individually on each sample. Whole genome libraries were prepared using the NEBNext Ultra II FS DNA library prep kit for Illumina. Extracted DNA was concentrated from 30 to 8 μL using a SpeedVac concentrator. A master mix was made with 0.4 μL of NEBNext Ultra FS Enzyme Mix and 1.4 μL of NEBNEXT Ultra FS reaction buffer for every DNA sample. 5.2 μL of the concentrated DNA was mixed with 1.8 μL of the master mix in 0.2 mL tubes. These were then pipette mixed and flash spun. The tubes were placed into a thermocycler to digest at 37°C for 8 min, followed by 65°C for 30 min. Samples were kept on ice when the digest cycle was completed.

A ligation master mix was made of 6 μL NEBNext Ultra II Ligation Master Mix and 0.2 μL NEBNext Ligation enhancer. Each mix was pipette mixed and flash spun down. The 7 μL sample digests were mixed with 6.2 μL of the ligation master mix and 0.5 μL of NEBNext Adapter for Illumina (5‐fold diluted to create a working adapter concentration of 3 μM). Each sample mix was pipette mixed and flash spun before being incubated at 20°C for 15 min in a thermocycler with the heated lid off. With the tubes placed on ice, 0.6 μL of USER was added to the ligations, pipette mixed and flash spun. The ligation mixtures were then incubated at 37°C for 15 min with the lid down and set to above 47°C.

Before PCR, the ligation mixtures were cleaned using SPRI beads. 0.8× SPRI beads (11 μL for 13.7 μL ligated DNA) were added, pipette‐mixed 10 times and incubated for 5 min. The tubes were then placed on a magnetic stand and left until the solution became clear. While still on the magnetic plate, the supernatant was removed, leaving the beads. The beads were then washed twice using 200 μL of freshly prepared 75% ethanol and left to dry for 5–10 min (until the beads are matte but not yet cracked). The beads were then eluted in 11 μL of water, pipette‐mixed and incubated for 10 min. The tubes were then placed onto a magnetic stand again and left until clear. 5 μL of each mixture was then transferred into two 96‐well plates.

The 5 μL of cleaned ligation products were mixed with 5 μL of NEBNext Ultra II Q5 Master Mix and 0.6 μL of barcoded adapters for Illumina. Before use, i5 and i7 oligos were combined and diluted to 5 μM. Each sample was labelled with two unique barcoded adapters. The plate containing the barcodes was spun down at 2,250 x g rpm prior to mixing. All samples were pipette mixed and flash spun down before being placed in the thermocycler for PCR enrichment: initial 98°C denaturation for 30 s; 10 cycles of 98°C denaturation for 10 s followed by annealing/extension at 65°C for 75 s; and a final extension at 65°C for 5 min.

Individual libraries were purified using a selective bead clean‐up, following the same 0.8x SPRI bead buffer exchange procedure described above. This step is aimed at decreasing the primer dimer whilst retaining the library, thus size‐selecting the libraries over 200–300 bp. In the final step, 11 μL of each sample was transferred to a new plate. 1 μL of each library was then quantified using the Quant‐IT dsDNA Assay high sensitivity kit and a Qubit fluorometer.

#### 2.3.3 Library Pooling and Sequencing

Individual barcoded libraries were pooled so that each library contributed 10 ng DNA, based on the Qubit quantification. A final 0.8× SPRI bead clean‐up of the whole pool was performed before sequencing. Fragment size distribution of the pool was measured using the Agilent Bioanalyzer System (Figure S2). All libraries were sequenced on a single S4 lane of an Illumina HiSeq 4000 by the University of Liverpool's Centre for Genomic Research (CGR). Raw Fastq files were trimmed by the CGR for the presence of Illumina adapter sequences using Cutadapt (v1.2.1) (Martin 2011) with the option –O 3 and further trimmed using Sickle (v1.2) (Joshi and Fass 2011) with a minimum window quality score of 20. Reads shorter than 15 bp were also removed by the CGR after trimming.

#### 2.3.4 Mapping, SNP Calling and Filtering

Trimmed reads were mapped to the *C. palaemon* reference genome (Lohse et al. 2023) using BWA‐MEM (v 0.7.12‐r1039) (Li 2013) and Samtools to sort the SAM files and convert them into BAM format. We used Picard Tools MarkDuplicates (Broad Institute 2019) to identify and then remove any duplicate reads. We used Samtools flagstat to assess the mapped reads, removing 3 samples from further analysis due to poor mapping. We performed SNP calling using BCFtools (v1.9) (Danecek et al. 2021) mpileup function. The called raw SNPs were filtered to remove: (1) indels; (2) loci with minor allele frequency (MAF) < 0.05, base quality < 30, mean depth < 10× or > 20× (averaged over all individuals), > 2 alleles, mapping quality < 30, and deviating from Hardy–Weinberg equilibrium at *p* > 0.05 for each sampling site; (3) individual samples with missing data > 50% or with < 3.0× average coverage (4 individuals removed from analysis); and (4) loci with missing data in more than 5% of the samples. The SNPs were also pruned to account for linkage disequilibrium (LD) using *r* ^2^ > 0.8 in 100 kb windows using BCFtools (Danecek et al. 2021). As the sample is a mixture of heterogametic (ZZ) males and homogametic (WZ) females, all SNPs on the Z chromosome were excluded from all analyses. Following filtering, we had a dataset of 114 individuals and 818,520 SNPs.

In addition, we created a dataset of mitochondrial DNA (mtDNA). We used NovoPlasty (v4.3.1) (Dierckxsens, Mardulyn, and Smits 2016) to assemble the mitochondrial genome of each individual with either the DToL mitochondrial genome or individual 81's mitochondrial genome as a seed (Lohse et al. 2023).

### 2.4 Analysis of *Carterocephalus palaemon* Population Structure

The nuclear SNP dataset was first subjected to a principal components analysis (PCA) to establish the population structure of the Belgian subpopulations and the location of the Fineshade within this structure using PLINK (v1.9) (Purcell et al. 2007) and R package PCAdapt (v4.3.3) (Privé et al. 2020). We then used ADMIXTURE (v1.3.0) (Alexander, Novembre, and Lange 2009) to infer individual ancestries from the SNP dataset from *K* = 1 to *K* = 5, running it with a 5‐fold cross‐validation. We established the optimal number for K using the lowest cross‐validation error and using evaladmix (Garcia‐Erill and Albrechtsen 2020) to evaluate the fit of the admixture under different K. To quantify the degree of nuclear genome differentiation, we calculated *F*_ST_ between the two Belgian landscapes and between individual subpopulations (including Fineshade) using the R package PopGenome (v2.7.5) (Pfeifer et al. 2014).

A phylogenetic tree of the mitochondrial genomes was reconstructed to gain further insight into the genetic structure of the Belgian subpopulations and the survival of matrilines through the reintroduction bottleneck at Fineshade. We used MAFFT (v7.515) (Katoh and Standley 2013) to align mitochondrial sequences (15, 728 bps) and IQtree (v 2.2.0.3) (Nguyen et al. 2015) with model selection (selected model: HKY + F + I, chosen according to BIC) and 1000 fast bootstrap to construct a maximum likelihood tree. Any individuals who did not pass the composition chi‐squared test run by IQtree were removed, leaving a subset of 117 individuals. The mitochondrial genome of the Scottish individual (Lohse et al. 2023) was used as the technical outgroup. To visualise the tree, we used FigTree (v1.4.4) (Rambaut 2018).

### 2.5 Estimation of Effective Population Sizes

We used the LD‐based method GoNe (Santiago et al. 2020) to calculate effective population sizes (*N*_e_). In the absence of a recombination map, the average recombination rate (*r*) was estimated by assuming a per‐chromosome map length of 50 cM (corresponding to a single cross‐over event per male generation). This assumption is consistent with empirical studies in other Lepidoptera (Nasvall et al. 2023). A weighted genomic average *r* of 4.2 cM/Mb was calculated using the length distribution of the 31 chromosomes (Lohse et al. 2023).

Effective population sizes were estimated separately for each of the two landscapes sampled in Belgium (Fagne and Famenne) over the past 100 generations, and henceforth we use the term “population” to denote this grouping level. Confidence intervals of the estimates were obtained by running 20 replicates, each one corresponding to a random sample of 2000 SNPs from each of the 30 autosomes. This procedure balanced information across chromosomes whilst minimising overlap of loci used among replicates (approximate range of percentage overlap between replicates across chromosomes 4%–17%). The same method was used to estimate the effective size of the population bottleneck during the establishment of the Fineshade population.

### 2.6 Dispersal Distances

Mean dispersal distances were calculated by using comparisons between genetic distance (*F*_ST_) and geographic distance (km) between subpopulations, or isolation by distance (IBD) (Rousset 1997; Wright et al. 2015; François et al. 2021; Smith and Weissman 2023). *F*_ST_ was calculated between each subpopulation as stated above. We estimated dispersal distances for three sets of genetic and geographic distances: Fagne and Famenne separately and the two landscapes combined to represent the whole region. Advantages and drawbacks of using the full set of subpopulations versus one landscape are discussed in Appendix S1.

The standard deviation of the dispersal kernel (σ), or dispersal kernel spread, can be calculated as *σ* = √(1/(4*πDb*)), where *b* is the slope of the linear regression between *F*_ST_ and geographic distance and *D* is the effective density of reproducing individuals (Flipovic et al. 2020; Rousset 1997). The effective density *D* is defined as *N*_*e*_/study area (two‐dimensional model) or *N*_*e*_/study area length (one‐dimensional model), where *N*_*e*_ is the effective population size (calculated as above; see also Figure S3). *N*_*e*_ of the Fagne and Famenne populations were added together to estimate *N*_*e*_ for the whole region. As the network of suitable habitat in our study is fairly narrow and elongated (Figure 1), the study area was treated as a linear habitat defined by the western and eastern extremities of the sampled sites, measured with ArcGIS (Fagne: 18 km; Famenne: 14.8 km; Fagne‐Famenne combined: 70.6 km). In accordance with a one‐dimensional model (Rousset 1997), we calculated the slope of the linear relationship between 1/(1 − *F*_ST_) and the untransformed geographic distance between sites in km. The slope was estimated using the lm() function in R (v4.0.2, R Core Team 2020).

We transformed the dispersal kernel spread into mean dispersal distances following two kernel types: the Laplacian kernel and the Gaussian kernel (Pinsky et al. 2017). Mean dispersal distances with Laplacian and Gaussian kernels were calculated using *σ*/√2 and *σ*√(2/*π*), respectively. Confidence intervals were calculated by using the upper and lower limits of *N*_*e*_ and the 95% confidence intervals of the regression slope.

### 2.7 Genetic Diversity Measures and Quantification of Inbreeding

Heterozygosity (observed and expected) and genome‐wide deviation from Hardy–Weinberg equilibrium (*F*_IS_) within each population were calculated using VCFtools het (v0.1.16) (Danecek et al. 2011). Runs of homozygosity (ROH) were analysed using PLINK (Purcell et al. 2007). We first re‐filtered the SNP loci without MAF or LD filtering. We then performed ROH analysis on all individuals, initially setting the minimum length of ROH to 500 kb, to calculate the number and length of ROH across populations. We repeated the analysis at a higher minimum length of 1 Mb. Significant differences in estimates of ROH between populations (Fagne, Famenne, and Fineshade) were evaluated using a Kruskal‐Wallis test with a Bonferroni‐adjusted *p*‐value using the outputs calculated with the minimum length set to 500 kb. A measure of individual autozygosity (fROH) arising from recent inbreeding was calculated by dividing the total ROH length (segments larger than 500 kb) by the estimated autosomal genome size of 375.9 Mb (Lohse et al. 2023; McQuillan et al. 2008). In all of these analyses, individuals were assigned to populations based on their geographic origin, rather than genetic assignment (McQuillan et al. 2008).

We used VCFtools (v0.1.16) (Danecek et al. 2011) relatedness 2 statistic to compute pairwise relatedness statistics between every pair of individuals within the dataset. This statistic is based on the kinship coefficient between each pair of individuals, which is the probability that any two alleles selected randomly from the same locus are identical by descent. We then used R packages ggplot2 (v3.4.2) and reshape2 (v3.6.3) (Zhang 2016) to visualise relatedness between the individuals in the Fineshade population on a heatmap.

### 2.8 *Wolbachia* Presence and Phylogeny

The standard DToL cobiont pipeline identified the presence of *Wolbachia* in the reference *C. palaemon* individual from Scotland. *De novo* assembly of the HiFi reads (NCBI: ERR9793196) using hifiasm (v0.15‐r334) (Cheng et al. 2021) generated a *Wolbachia* complete genome (1.33 Mb) plus a second shorter (0.44 Mb) *Wolbachia*‐like scaffold. In order to focus on the primary *Wolbachia* genome and exclude paralogous sequences, we tested for the presence of *Wolbachia* in each *C. palaemon* individual by aligning the reads competitively using Bowtie 2 (Langmead and Salzberg 2012) against both scaffolds, as well as the mitochondrial reference, producing fastq files for each individual. We then used SAMtools (Danecek et al. 2021) to establish the breadth of coverage of the *Wolbachia* reference from each sample to indicate the presence of *Wolbachia* within the Famenne and Fagne landscapes and the Fineshade population. A cut‐off for confirmed presence within an individual was 50% coverage.

*Wolbachia* SNPs were called from the individual fasta files using BCFtools (v1.9) (Danecek et al. 2021) mpileup function using the concatenated *Wolbachia* + mitochondrial + *Wolbachia*‐like scaffolds as the reference. The raw SNPs were filtered for quality and depth as per the nuclear genomes. This left a dataset of 118 individuals and 2454 SNPs. We then used MAFFT (v7.515) (Katoh and Standley 2013) to align the sequences and IQtree (v 2.2.0.3) (Nguyen et al. 2015) with model selection (selected model: HKY + F + I, chosen according to BIC) and 1000 fast bootstrap to construct a maximum likelihood tree, removing any individuals that did not pass the composition chi‐square test run by IQtree. This left a subset of 117 individuals. We used the *Wolbachia* genome established from the reference *C. palaemon* individual from Scotland as the technical outgroup for the phylogenetic tree. To visualise the tree, we used FigTree (v1.4.4) (Rambaut 2018).

## 3 Results

### 3.1 Population Structure

Population structure and coancestry were revealed by PCA and admixture analyses of the nuclear genome SNPs and by resolving the mitochondrial genome phylogeny, with all three approaches giving concordant results to our first two research questions. The Belgian individuals form two clearly disjoint genetic clusters, corresponding to the Fagne and Famenne landscapes (map in Figure 1), with little differentiation between the sampled sites within each landscape. So, henceforth, we focus on reporting whether individuals are Fagne‐like or Famenne‐like and term these “populations”, although site (“subpopulation”) labels are still shown. Placement of individuals into groups within the overall population structure based on each analysis is shown in Table S2; this table can be used to cross‐reference the individual codes used throughout.

**FIGURE 2:**
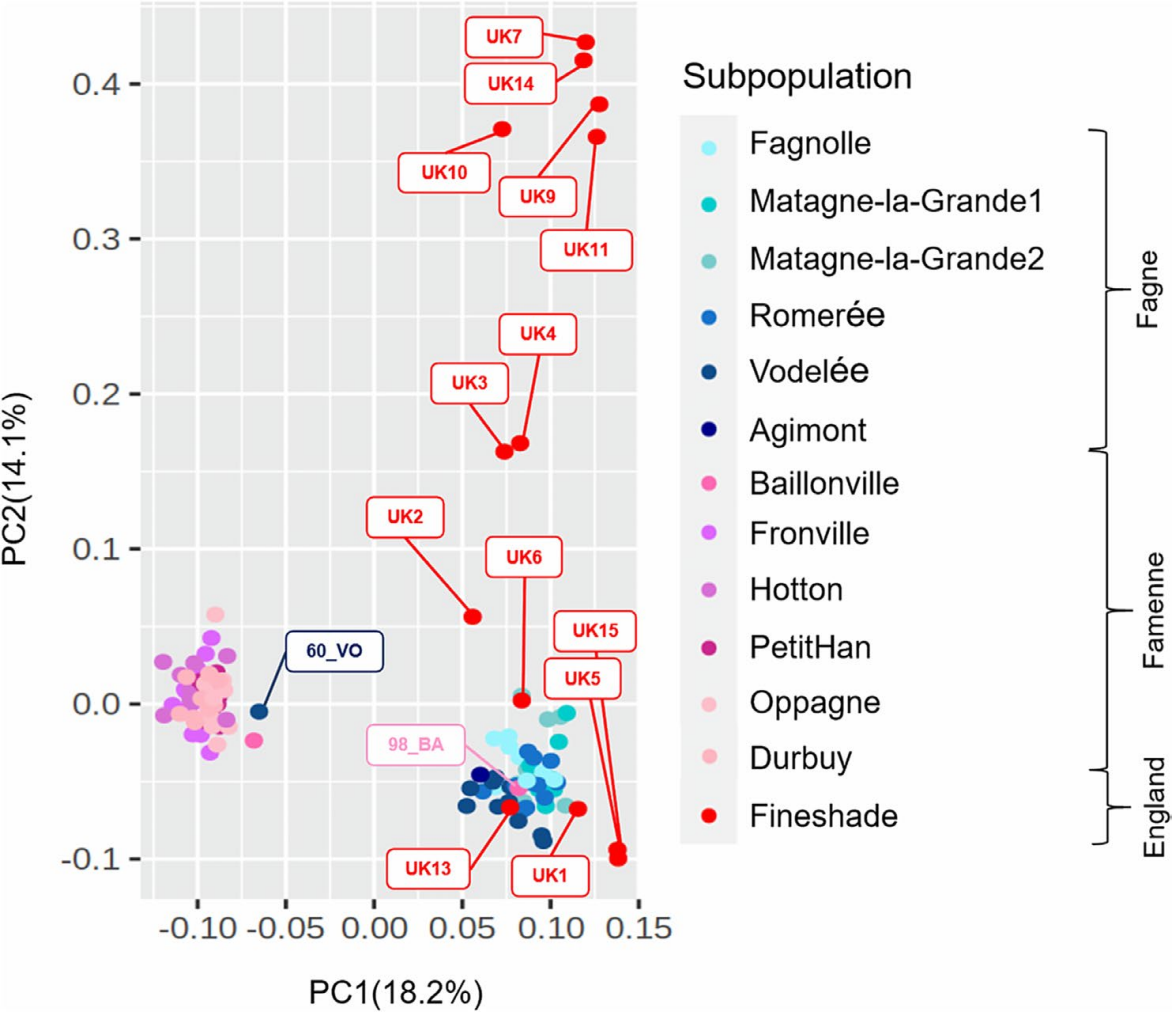
Genetic population structure of *C. palaemon* in Belgian (source) and Fineshade (reintroduced) populations. Principal Components Analysis of genetic distances between all individuals based on whole nuclear genome SNPs, calculated in PCAdapt with *k* = 13. Circles on the PCA indicate the position of individual genotypes on the first two principal component axes, coloured according to collection site and landscape: Shades of blue (Fagne, source); shades of pink (Famenne, source), and red (Fineshade, reintroduced). Individual identifying labels are added for the 13 Fineshade individuals and for two Belgian individuals that are genetically mismatched to their geographic locations.

The Fagne‐Famenne split is very evident in the first axis of the PCA, which explains 18.2% of the variance (Figure 2). There are two Belgian individuals that appear on the PCA to be genotypically mismatched with their landscape provenance. These individuals come from the subpopulations of Vodelée and Baillonville (individual codes 60_VO and 98_BA, labelled on Figure 2), that lie at the eastern and western limits of the sampled landscapes, respectively (Figure 1), which suggests that they may be recent migrants. On PC1, all of the Fineshade individuals (descendants of the reintroduction to England) are clearly closer to the Fagne than the Famenne cluster. PC2 (explained variance 14.1%) contrasts the Fineshade individuals against the Belgian samples, with the large spread of Fineshade consistent with genetic drift caused by a bottleneck event.

**FIGURE 3:**
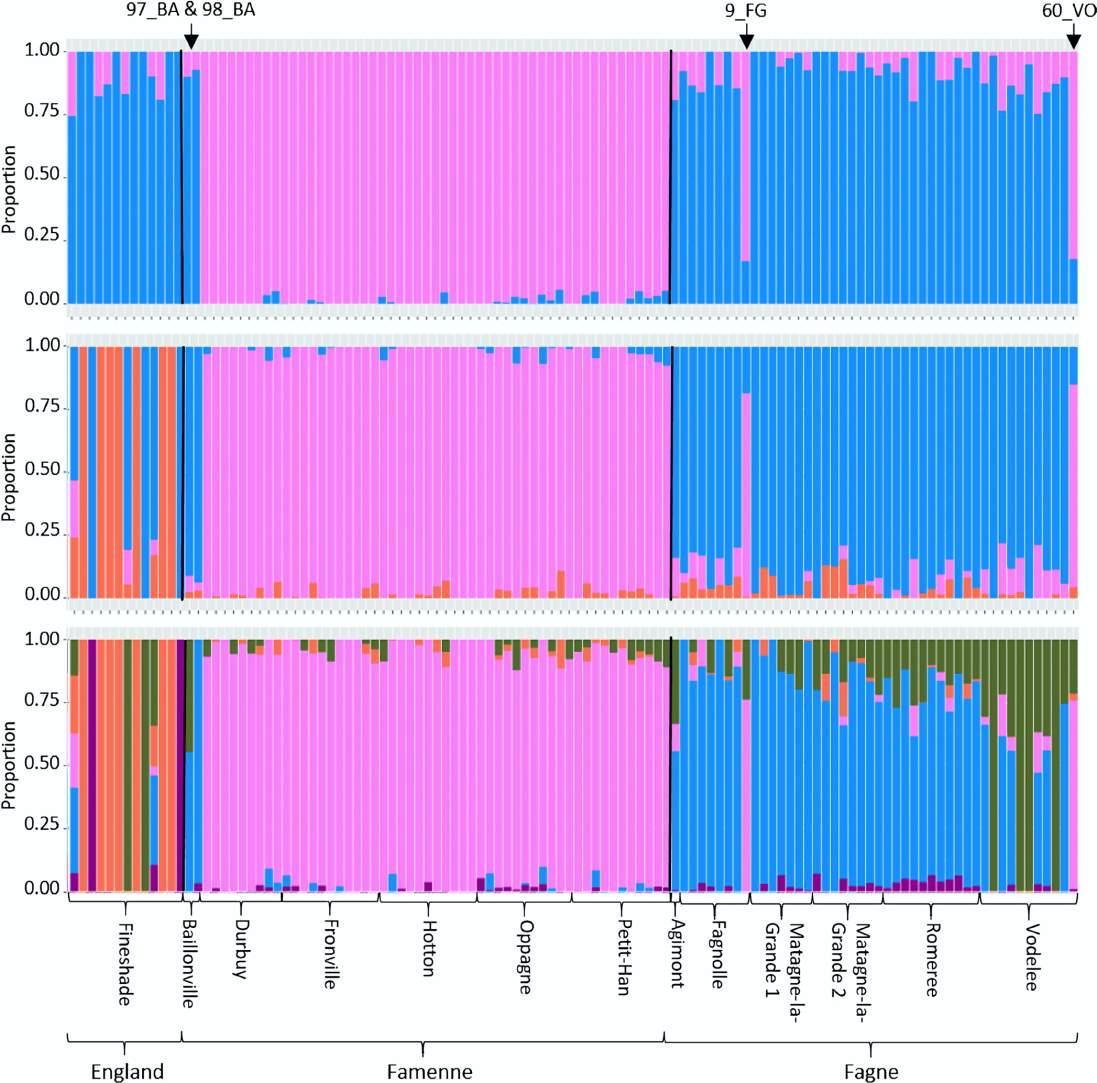
Coancestry among *C. palaemon* individuals in Belgian (source) and Fineshade (reintroduced) populations. Admixture plots showing the genetic ancestry of each individual, splitting the genome into groups represented as colours based on this inferred ancestry. Each bar represents a single individual and the same colour grouping represents similarity in genetic ancestry. Results are shown for different numbers of ancestral populations (*K* = 2, 3 and 5 from top to bottom), but *K* = 2 has the lowest cross‐validation error. Individuals that are genetically mismatched to the landscape where they were sampled are labelled at the top.

Population structure according to the admixture analysis (Figure 3) also showed a geographic split between Famenne and Fagne landscapes in Belgium, and Fineshade individuals were shown to be much more closely related to the Fagne individuals. The most strongly supported number of ancestral populations (K) is two (see pink and blue colours at the top of Figure 3), corresponding to the Fagne‐Famenne split in Belgium, with all Fineshade individuals resembling the Fagne profile. However, there is an indication of a small amount of geographic exchange between the Famenne and Fagne landscapes (9_FG, 97_BA, 98_BA and 60_VO). Two of these seem to be clear recent immigrants (98_BA and 60_VO), as all analyses agree on their placement (Table S2). The four mismatched individuals retain their contrasting ancestry profiles at all levels of *K*. The additional groups that appear at *K* = 3 and *K* = 5 are mainly represented by individuals from Fineshade, which is consistent with some of the large genetic distances shown on the PCA. The higher levels of K generally do not reveal additional substructure in the Belgian subpopulations, although at *K* = 5, Vodelée contains a higher representation of the green group (Figure 3).

**FIGURE 4:**
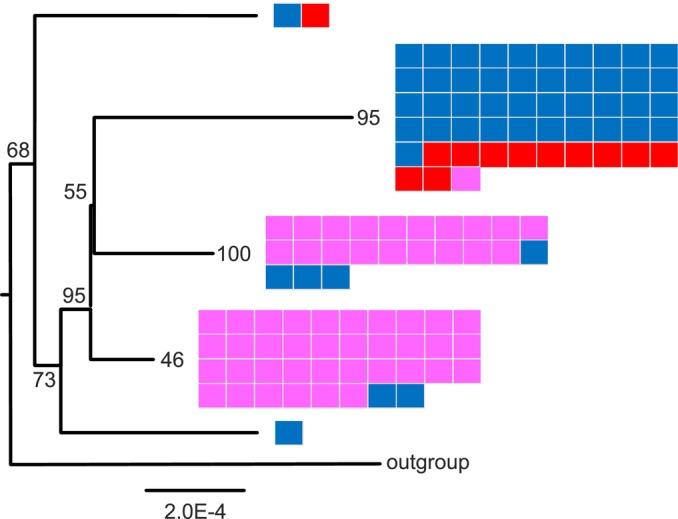
Simplified phylogenetic tree of Belgian (source) and Fineshade (reintroduced) *C. palaemon* complete mitochondrial genomes. The deepest branches of the maximum likelihood tree are shown, and the full tree with individual labels is given in Figure S6 Small boxes are shown to represent the individuals occurring on each branch, coloured by the geographic origin landscape: Fagne (blue), Famenne (pink), or Fineshade (red), and these are ordered by landscape, not by their position in the subtrees. The reference genome individual from Scotland was used as the technical outgroup. Numbers are bootstrap support.

The phylogenetic tree of the mitochondrial DNA (Figure 4) places the majority of the individuals into three main clades: one majority Fagne and two majority Famenne. Compared with the nuclear genotypes, there is more crossover of mitotypes from the landscapes in Belgium into clades with majority individuals from the opposing landscape (55 & 60_VO, 21,22 & 26_MG2, 3_FG and 98_BA; see Figure S6 and Table S2). All of the reintroduced Fineshade samples are in the Fagne clade, except for one individual (UK6) that is placed on a peripheral branch with a single Fagne individual (20_MG2; see the top branch of Figure 4 and the labelled tree Figure S6).

There is a maximum *F*_ST_ of c. 0.047 between individual Belgian subpopulations (Figure S5) and an *F*_ST_ of 0.043 between the Fagne and Famenne populations (when all subpopulation data is combined). Pairwise *F*_ST_ between Fineshade and each Belgian site reinforces the result that the reintroduced population is genetically closer to the Fagne than Famenne populations (Table 2). Matagne‐La‐Grande 1 and Matagne‐La‐Grande 2, the two sites from which the most individuals were sourced for the reintroduction (Table 2), showed the lowest pairwise *F*_ST_ with Fineshade.

**TABLE 2:** *F*_ST_ between the reintroduced Fineshade *C. palaemon* population and each sampled Belgian subpopulation in the source region.

Site in Belgium	Landscape	*F*_ST_ compared to Fineshade	# of individuals taken for reintroduction, by year (female, male)	
			2018	2019
Fagnolle	Fagne	0.042	0	0
Matagne‐La‐Grande2	Fagne	0.023	12 (9, 3)	7 (3, 4)
Matagne‐La‐Grande1	Fagne	0.035	12 (10, 2)	7 (4, 3)
Romeree	Fagne	0.045	0	0
Vodelée	Fagne	0.043	5 (5, 0)	0
Fronville	Famenne	0.061	5 (4, 1)	10 (5, 5)
Hotton	Famenne	0.077	0	0
Petit‐Han	Famenne	0.072	0	0
Oppagne	Famenne	0.068	6 (4, 2)	0
Durbuy	Famenne	0.068	2 (0, 2)	0

*Note:* The number of adult females and males from each site used for the reintroduction into Fineshade is also given.

### 3.2 Isolation by Distance and Dispersal Capacity

Pairwise *F*_ST_ among Belgian sites increases with geographic distance (Figure S5). This was supported by the linear model, which showed geographic distance significantly predicted 1/(1 − *F*_ST_) (*R* ^2^ = 0.81, *p* < 0.001 when all sites are included). To address our third research question, we estimated mean dispersal distances (Rousset (1997)), informed by the contemporary estimates of *N*_*e*_ for the Fagne and Famenne populations (Figure S3). The mean dispersal distances based on either Fagne or Famenne data separately were approximately 1.2 or 1 km, respectively (Table S1). However, these estimates lack statistical power (see Appendix S1). When all sampled subpopulations from Belgium were analysed together (i.e., 10 sites, 45 pairs, Figure S5), we obtained higher mean dispersal distances of approximately 2.6 km and 4.6 km for Gaussian and Laplacian kernels, respectively. This analysis also has a notable limitation (see Appendix S1): the assumptions of the analysis will be seriously violated if there is any reduced fitness of Fagne‐Famenne hybrids (see sections below). Despite the uncertainties, the isolation by distance analysis gives a plausible range for the dispersal distances that would be consistent with the genomic data.

### 3.3 Genetic Bottleneck, Diversity and Inbreeding Associated With Reintroduction

The genetically effective population size of the reintroduced Fineshade population was estimated to be 32.7 (95% CI 32.5–33.0), and this estimate was constant for the four post‐reintroduction years. This size is consistent with the numbers of individuals released over two seasons (see Table 2). The released females were assumed to have been previously mated (based on the time of year that they were collected), each carrying the equivalent of two diploid genomes. Given that the surviving population seems to be exclusively descended from Fagne individuals, an *N*_e_ of 33 implies that the reproductive success of these founders was not strongly skewed (approx. 69 founder genomes, assuming 31 singly mated females over two years, and counting the 7 males in 2019 but not the 2018 males). The reconstructed *N*_*e*_ of the Fineshade population in the years before reintroduction (Figure S4) appeared larger than our estimate of the recent *N*_*e*_ of the Fagne landscape (Figure S3), but this inconsistency may be a side effect of the small Fineshade sample size (*n* = 13) combined with their genetic heterogeneity (Figures 2 and 3).

Genetic diversity, as measured by observed and expected heterozygosity (Table 3), did not differ significantly among the reintroduced (Fineshade) and source populations (Fagne and Famenne). *F*_IS_ estimates (Table 3) are consistent with low levels of inbreeding in all three populations and low levels of population structure within the more spatially extended Belgian populations, particularly Fagne.

**TABLE 3:** Genetic diversity and inbreeding statistics for Belgian (source) and Fineshade (reintroduced) populations of *C. palaemon* .

Population	*H*_o_	*H*_e_	*F*_IS_	#ROH (> 500 kb)	#ROH (> 1 Mb)	ROH length (Mb)	fROH (> 500 kb)
Fagne (source) *n* = 45	0.260 (0.256–0.263)	0.267 (0.263–0.270)	0.026 (0.013–0.039)	5 (0–43)	1 (0–9)	627	0.010 (0.008–0.013)
Famenne (source) *n* = 56	0.262 (0.259–0.266)	0.267 (0.263–0.270)	0.017 (0.002–0.031)	7 (0–37)	1 (0–5)	626	0.015 (0.011–0.019)
Fineshade (reintroduced) *n* = 13	0.263 (0.255–0.271)	0.266 (0.259–0.274)	0.013 (−0.041–0.041)	3 (0–14)	1 (0–3)	2483	0.018 (0.003–0.032)

*Note:* Means and 95% confidence intervals of observed (*H*_o_) and expected (*H*_e_) heterozygosity and within‐population inbreeding coefficient *F*_IS_. Median and range for the total number of runs of homozygosity (ROH) per individual > 500 kb and 1 Mb. Mean length of ROH segments above 500 kb. And the proportion of the genome in ROH > 500 kb (fROH) with 95% confidence intervals.

Additionally, there was no difference across populations in the average number of homozygous haplotypes (ROH) segments above 500 kb per individual in each population (*p* > 0.05), indicating low levels of ROH across all populations (Table 3). The mean length of ROH was higher in the Fineshade population as compared to the two source populations, inflated by very long runs in two Fineshade individuals, UK5 and UK15, which had average ROH lengths of 13,744 kb and 12,610 kb, respectively. However, all other individuals from Fineshade had average ROH lengths ranging from 525 kb to 925 kb, which was consistent with the mean lengths from the source populations (see Table 3). The probability of autozygosity (fROH) at the 500 kb scale across the genome had a slightly higher mean in Fineshade but a very wide confidence interval (Table 3). These results indicate that the reintroduced population has so far experienced minimal levels of inbreeding owing to relatively high haplotype diversity in the founder gene pool and fairly even reproductive success.

**FIGURE 5:**
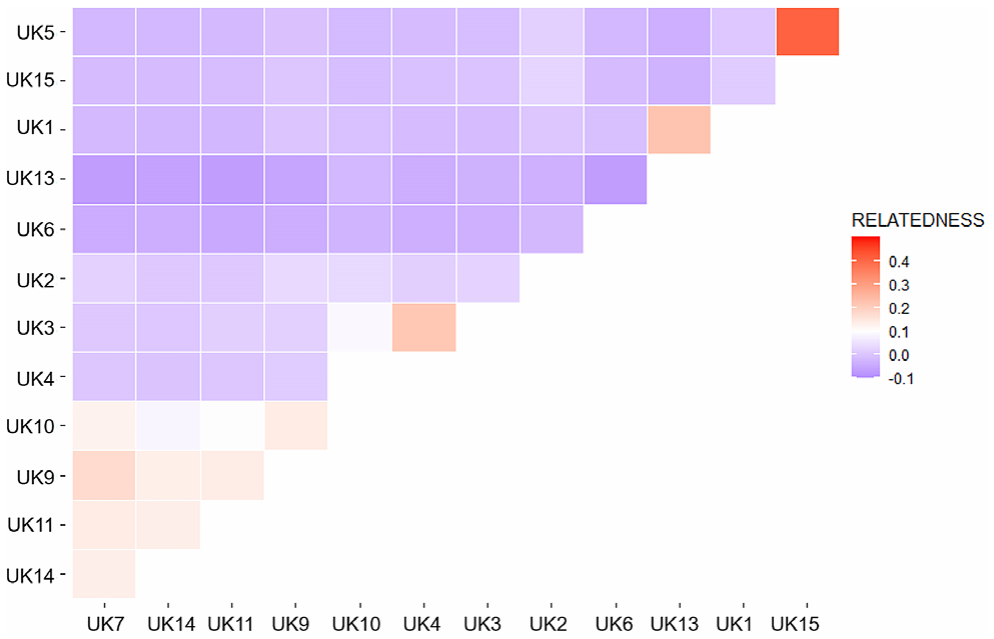
Pairwise relatedness between all *C. palaemon* individuals in the reintroduced Fineshade sample. The relatedness scale is based on the kinship‐coefficient between each pair of individuals, which is the probability that any two alleles selected randomly from the same locus are identical by descent. Purple tones indicate not different from zero, white/pale tones indicate a close relationship (ranging from close cousin to sibling), and darker/red tones are highly related pairs where prior inbreeding has occurred.

High levels of relatedness were found between some individuals within the Fineshade population sample (Figure 5). The most extreme example of this is seen between individuals UK5 and UK15, who appear to be siblings with parents who were also siblings. These levels of relatedness were not observed in the source populations, where the percentage of pairs that were either related or highly related was much lower (Table 4).

**TABLE 4:** Degree of relatedness among pairs of *C. palaemon* individuals within Belgian (source) and Fineshade (reintroduced) populations.

Landscape	Percentage of pairs in each kinship category (range of kinship coefficient)		
	Unrelated (< 0.1)	Related (0.1–0.2)	Highly related (> 0.2)
Fineshade (Reintroduced)	80.78	14.10	5.12
Fagne (Source)	96.41	3.58	0
Famenne (Source)	99.93	0.06	0

*Note:* The percentage of individuals sampled that were either related or highly related was much higher among pairs of individuals from Fineshade than the source landscapes.

### 3.4 *Wolbachia* Presence and Phylogeny

**FIGURE 6:**
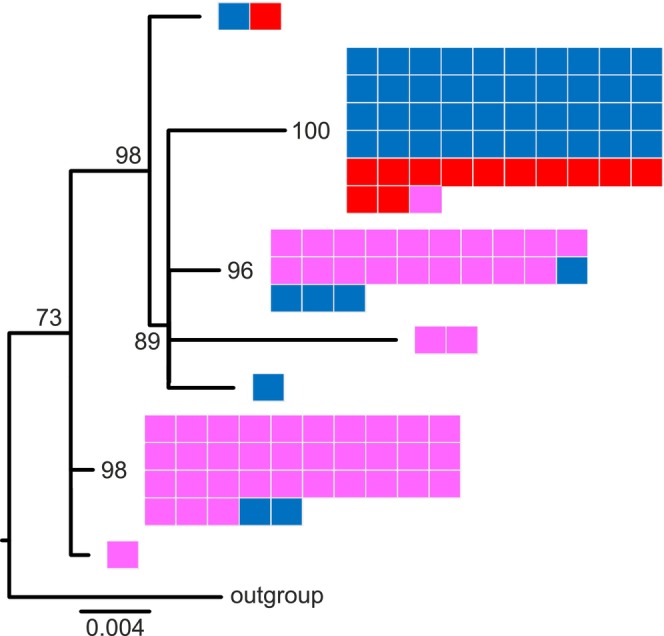
Simplified phylogenetic tree of *Wolbachia* detected in *C. palaemon* from Belgian (source) and Fineshade (reintroduced) population samples. The deepest branches of the maximum likelihood tree are shown, and the full tree with individual labels is given in Figure S7. Small boxes are shown to represent the individuals occurring on each branch, coloured by the geographic origin landscape: Fagne (blue), Famenne (pink), or Fineshade (red), and these are ordered by landscape, not by their position in the subtrees. The *Wolbachia* from the reference genome individual from Scotland was used as the technical outgroup. Numbers are bootstrap support.

The average breadth of coverage of the *Wolbachia* reference sequence was 50.11% (range 1.3–96.5) in Famenne individuals, 66.2% (20.1–97.6) in Fagne individuals, and 90.3% (74.3–97.8) in the Fineshade individuals. This shows the presence of *Wolbachia* in all populations and the majority of individuals sampled; the small number (18) in which *Wolbachia* was not conclusively detected is likely due to limitations of wing‐clip sampling. The phylogenetic tree of the *Wolbachia* essentially recapitulates the host mitochondrial genome tree, with three main clades: one majority Fagne and two majority Famenne (Figure 6). Geographic origin and *Wolbachia* clade assignment are mismatched for a few individuals, predominantly geographically Fagne individuals falling within Famenne‐type *Wolbachia* clades. All the *Wolbachia* group within the equivalent clade of their host's mitochondrial genome in the mitochondrial tree, with the exception of two samples (133_HO and 165_PH; compare Figures S6 and S7 and see Table S2), which group together on peripheral branches of different Famenne clades. Of the 13 Fineshade individuals (out of 15 sampled) for which *Wolbachia* status could be determined, 12 fall within the main Fagne clade, and one (UK6) pairs with a single Fagne individual (20_MG2) on a peripheral branch (Figure S7).

## 4 Discussion

We aimed to evaluate the genetic success of the recent reintroduction of *C. palaemon* to England by comparing genomes of the reintroduced population with those in the source region in Belgium. Two translocations have taken place to Fineshade Wood, plus an additional translocation to a second site in the Rockingham Forest landscape. Future genetic reinforcement or introduction to additional English sites is under consideration. Therefore, we highlight where our results can guide future strategy, as well as comment on the potential for using similar genomic techniques in other species.

### 4.1 Genetic Structure of the Donor Populations

We found unexpectedly strong genetic structuring of *C. palaemon* within a relatively small (80 × 50 km) region in south Belgium: one cluster associated with the Fagne landscape and the other with the Famenne landscape. The distinctness of the genetic clusters, i.e., the absence of any intermediate individuals, is unexpected given that the two landscapes have similar geology, altitude, habitats and phenology, and given the absence of major barriers to dispersal. It is believed that *C. palaemon* uses the same larval host plant in Fagne and Famenne ( *Brachypodium sylvaticum* ), although in general the species is oligophagous (Moore 2004). The concordance of these clusters between the nuclear and mitochondrial genomes (Table S2) suggests historical and perhaps ongoing barriers to gene flow. However, it is difficult to reconcile the different lines of evidence that might indicate the frequency of dispersal and successful mating over the ~50 km gap between the landscapes.

Firstly, the location of two individuals (60_VO and 98_BA, see Table S2) is consistently mismatched with all four genetic analyses, and thus they appear to be recent inter‐landscape migrants, implying that there are no absolute barriers to immigration in either directions. Secondly, under the strong assumption that the system is in migration‐drift equilibrium (Whitlock and McCauley 1999) the level of genetic differentiation between the Fagne and Famenne clades (*F*_ST_ ~ 0.04) implies that, on average, the populations exchange 6 migrants per generation, which represents 0.5% of each population (Appendix S1). Despite the uncertainty in our *F*_ST_‐based dispersal distance estimates (Appendix S1) (Bohrer, Nathan, and Volis, 2005; Petrovski and Morozov 2009; Petrovski, Morozov, and Li 2008), it seems highly unlikely that 0.5% of each generation's population could travel > 50 km; rather, it is more reasonable to assume that the exchange occurs over several generations, using populated stepping stones. There are additional subpopulations lying between the two sampled landscapes, where we could not ascertain the genetic profile (Figure 1). Nevertheless, we would expect any regular migration via such stepping stones to result in genetically intermediate individuals. In contrast, of the Belgian individuals whose nucleus and mitochondria could both be genotyped (*n* = 101; Table S2), none show intermediate nuclear genotypes; two have an admixture assignment that is inconsistent with PCA and mitotype (individuals 9_FG and 97_BA, see Table S2), and three have inconsistent nuclear vs. mitotype assignments (individuals 3_FG, 26_MG2 and 55_VO, see Table S2).

The reintroduction strategy of sourcing individuals from subpopulations across Fagne and Famenne was intended to maximise genetic diversity. This entirely reasonable approach would not necessarily have changed had prior knowledge of the regional population structure been available at the outset of the reintroduction programme, as there would have been no reason to suspect that individuals from one landscape would survive better than the other. Within each landscape, genetic admixture shows strong mixing between the subpopulations, so the sampling strategy within each landscape would not have affected reintroduction diversity. Thus, the use of locally observed abundance to guide sampling intensity among sites was appropriate, minimising impacts on the donor sites (Maes et al. 2019; Wildman 2023).

### 4.2 Genetic Character of the Reintroduced Population

The reintroduced Fineshade population appears to have descended exclusively from Fagne and not Famenne, ancestors, which implies that those introduced individuals were more successful at producing broods that survived. This conclusion is supported by all three analyses of the nuclear population structure (PCA, admixture and *F*_ST_), as well as the phylogenetic analyses of the mtDNA and *Wolbachia*. Although some of the Fineshade individuals have notably diverged from the typical Fagne genetic profile within a short time, there is no indication that this change was a result of Fagne‐Famenne hybridisation (the admixture plots would be most likely to show this process if it occurred). Therefore, although we cannot entirely rule out a small genetic contribution of Famenne individuals to the Fineshade population, it appears highly unlikely.

There could be several reasons for the higher breeding success of individuals from the Fagne landscape introduced to Fineshade. Firstly, about twice the number of adults released came from Fagne as from Famenne (43:23), which could have been further skewed by stochastic factors. The influence of numbers released on genetic contribution is further indicated by Fineshade's *F*_ST_ versus the Fagne sites, where the lowest values are for the two sites with the largest donor samples (Matagne‐La‐Grande 1 & 2). However, on its own the reduced initial input cannot account for the complete absence of Famenne genetic profiles in Fineshade. Furthermore, systematic differences in physical condition at the point of release are considered unlikely, because field collections from both donor landscapes took place simultaneously across 2 days each year, both sets of butterflies were kept at similar cool temperatures during travel, and all butterflies were visibly healthy at the time of release into Fineshade Wood (Bourn et al. 2024).

Alternatively, it could be that individuals from Fagne are more ecologically suited to the new site, for example, having slight differences in host plant or habitat/microclimate preferences or phenology (Houde, Garner, and Neff 2015). If so, this would have major implications for any future translocations to Fineshade and the surrounding landscape, as it would therefore be preferable to translocate individuals from Fagne subpopulations that would have a better chance of survival. Lastly, there could be some genetic incompatibility between individuals from the two clades that reduces the likelihood of inter‐clade offspring being produced and surviving.

The 2018/2019 reintroduction of *C. palaemon* has shown signs of success, with the population persisting and expanding in area occupied and estimated population sizes increasing (Wildman 2023). In addition, the reintroduction has been largely successful at retaining similar levels of genomic heterozygosity to those found in the Fagne and Famenne landscapes in Belgium. This has been achieved even though the founder pool was smaller than expected (as Famenne individuals left no descendants). Maintenance of genetic diversity in the initial years is one of the most important factors in reintroductions (Andersen et al. 2014), and therefore these results are promising for the population. The genetically inferred *N*_*e*_ of Fineshade (33) implies that approximately half of the 31 females (2018 and 2019) and 7 males (2019) introduced from Fagne reproduced successfully, reflecting the suitability of the restored habitat.

The restricted number of founders and variance in reproductive success among the surviving families in Fineshade has resulted in a higher proportion of closely related individuals than in the donor populations and a slightly higher inbreeding profile, as measured by fROH, but not detectably elevated homozygosity. The negligible effect on heterozygosity may be due to a lack of power to detect the relatively small reduction in heterozygosity expected in an idealised population of size (*N*_*e*_) 33 for three consecutive generations (*H*_*e*_ = 0.045; *H*_*o*_ = 0.03). However, it is also possible that the bringing together of two somewhat diverged Fagne subpopulations (Matagne‐La‐Grande and Vodelée) into Fineshade has partially counteracted the expected drift effect on homozygosity.

The high levels of inbreeding and kinship that we would expect to cause significant inbreeding depression for fitness (Saccheri, Brakefield, and Nichols 1996) are only shown for a small fraction of Fineshade individuals as of 2022. However, managers should remember that genetic diversity in small populations progressively erodes, so the initial situation can easily change. In the short to medium term, if *N*_*e*_ recovers quickly and stabilises above ~200 (representing several hundred adults), the expected rate of genetic drift and inbreeding (1/2*N*_*e*_; corresponding to 2.5% over 10 generations; Hartl 1988) will be low enough to retain most of the genetic diversity present in 2022 and avoid inbreeding depression (Jamieson and Allendorf 2012; Rosenfeld 2014). Conversely, if these levels of kin‐mating continue, heterozygosity will fall (Pekkala et al. 2014), and there could be longer‐term consequences for the population, such as the accumulation of deleterious alleles, the loss of potentially beneficial alleles, and the inability of the population to respond to changes in the environment (Vanden Broeck et al. 2017).

### 4.3 A Potential Role of *Wolbachia* in Host Divergence and Lineage Survival

The detection of *Wolbachia* in the DToL reference from Scotland and in a majority of the individuals in this study, and the strong congruence between mitochondrial and *Wolbachia* trees, indicate that they have been diverging together within *C. palaemon* for an extended period, likely in the order of thousands of generations (Jiggins 2003). Indeed, the distinct pattern of spatial divergence between the *Wolbachia* clades within the study region, in the absence of an obvious geographic boundary, together with the loss of Famenne‐type *Wolbachia* strains that must have been introduced with the translocated adults, might suggest an active role of the endosymbiont in the contrasting survival of founder lineages within the reintroduced Fineshade population. The most likely mechanism for such an effect is cytoplasmic incompatibility (CI), used by *Wolbachia* for promoting self‐transmission. Unidirectional CI happens when an infected male mates with an uninfected female, whereas bidirectional CI involves incompatibility between different strains of *Wolbachia* (Bordenstein and Werren 2007). Both types can lead to host embryo death, with varying degrees of leakage. Bidirectional CI between incompatible strains in the Fagne and Famenne clades could be preventing breeding between the clades: both the breeding of inter‐cluster migrants and breeding post‐reintroduction. We do, however have to acknowledge that we cannot confirm this hypothesis with our data alone, as we cannot establish that *Wolbachia* is the causal agent of the loss of Famenne individuals, as opposed to the Famenne‐type *Wolbachia* clade being lost together with the Famenne individuals for some other reason. Therefore, we suggest that the hypothesis should be further tested by cross‐breeding individuals from each clade in captivity and monitoring offspring survival rates.

### 4.4 Future Prospects for Chequered Skippers in England

The reintroduction of *C. palaemon* to Fineshade has been largely successful, thus far, in retaining high levels of genetic diversity. However, the measured *N*_*e*_ and kinship profile could eventually impact genetic diversity. Therefore, it would be beneficial to supplement the population further to minimise the rate of inbreeding in the future. If further research was feasible prior to making translocation decisions, it would be worth investigating whether Fagne individuals have a fitness advantage in England or whether individuals from the separated Belgian landscapes are incompatible. However, we would argue that there is a sounder rationale for using Fagne than Famenne individuals for further translocations based on our results alone.

The rationale is that the Fagne populations have proved to be most successful in Fineshade, so these are a less risky choice, no matter what caused the contrasting survival. Additional Fagne genotypes would still boost the English genetic diversity to some extent. Continuing to source from Famenne in the hope that this will be better for genetic diversity runs the risk of wasting effort and even harming the fitness of the reintroduced population if there is genetic incompatibility. Choosing a risk‐averse strategy is particularly important because translocating individuals can have an impact on the source population (Weeks et al. 2011), and so it is important to make the most efficient use of the donor individuals that are removed.

Translocations into a second site within the Rockingham Forest landscape, using the same source sites as the initial translocation into Fineshade Wood, took place from 2022 onwards. This could provide an interesting opportunity to examine the questions raised by the results of this study through further genetic testing. For example, if we were to find that individuals in this second reintroduced population were only descended from Famenne, this would render the genetic incompatibility hypothesis more likely than the hypothesis that Fagne individuals have an ecological advantage.

### 4.5 Utility of Non‐Destructive Sampling

This study has demonstrated major potential benefits of genomic analysis for reintroduction and genetic reinforcement projects. We have shown the significant amount of information that can be gained from the non‐destructive method of wing‐clipping while not significantly affecting the sampled populations to gain this information. Previously, wing‐clipping methods have been mostly restricted to mitochondrial (Lushai et al. 2000) or microsatellite analyses (Roland, Keyghobadi, and Fownes 2000; Saarinen, Austin, and Daniels 2010; Vanden Broeck et al. 2017; De Ro et al. 2021). While non‐lethal leg samples have previously been used for whole‐genome sequencing for insects (Gershman et al. 2021), we now show that sufficient DNA can be gained from even smaller amounts of tissue.

We also show the benefits of genetically testing populations both before and after reintroductions. This new knowledge highlights the importance of knowing the population structure in source populations, so decisions on how many source populations to use and the spatial scale in which to choose source populations can be better informed. Our study, together with other similar ones, could eventually be used to formulate practitioner guidelines for sampling to avoid outbreeding depression (of which genetic incompatibility is an extreme example). In this specific case, the source population genetic structure may not have influenced the initial sampling strategy, because the cytoplasmic incompatibility theory was only raised after the reintroduced population had established. However, this knowledge is likely to impact the strategy for any future translocations needed to boost the population. In addition, we show that genetically testing the reintroduced population, even very soon post‐release, can highlight potential issues such as inbreeding and can inform practitioners on best practices moving forward. It can inform on whether to proceed with any additional translocations, where to place any more translocated individuals (to boost initial populations or place individuals in new sites), and the best source sites to continue with (Houde, Garner, and Neff 2015). In general, reintroduction programmes operating in the absence of such genetic information have a lower probability of long‐term success (Davis et al. 2021). While it is important to acknowledge that the costs of genetic testing are high, they are not disproportionate in comparison to the overall costs of reintroductions and the associated habitat rehabilitation, which are at least thousands of Euros/Dollars per year (Hilbers, Huijbregts, and Schipper 2020). Therefore, we argue such spending offers good value in order to focus efforts and reduce the likelihood of failure.

## 5 Conclusions

The reintroduced *C. palaemon* population in Fineshade Wood, England, is likely descended from just one of the source landscapes in south Belgium (Fagne) but has nevertheless retained a high proportion of the genetic diversity. The genetic bottleneck experienced during the first 4 years post‐reintroduction is not a major concern to the population's persistence, but if similar rates of inbreeding and small *N*_*e*_ persist, inbreeding depression and/or loss of valuable genetic diversity could impact its growth rate and adaptability in the coming years. Based on the pattern of survival of Fagne over Famenne‐introduced lineages, a conservative strategy for any further translocations would prefer Fagne sites as sources, both for augmentation of Fineshade Wood and the colonisation of neighbouring sites. However, further investigation into the underlying causes of population structure in Belgium could lead to a different strategy.

## Conflicts of Interest

The authors declare no conflicts of interest.

## Supporting information


Data S1.

## Data Availability

Raw DNA sequences are available at the European Nucleotide Archive study accession PRJEB72533.
