# Supplementary material for: Genomic Monitoring of a Reintroduced Butterfly Uncovers Contrasting Founder Lineage Survival
Source: Evol Appl. 2025 Feb 6;18(2):e70074. doi: 10.1111/eva.70074 (PMC11802331; doi:10.1111/eva.70074)
Supplement: Supplementary file 1 — Data S1. [file EVA-18-e70074-s001.docx]

Supplementary material for: *Genomic monitoring of a reintroduced butterfly uncovers contrasting founder lineage survival*

Georgina Halford, Dirk Maes, Carl J. Yung, Sam Whiteford, Nigel A. D. Bourn, Caroline R. Bulman, Philippe Goffart, Jenny A. Hodgson & Ilik J. Saccheri. Evolutionary Applications, 2025


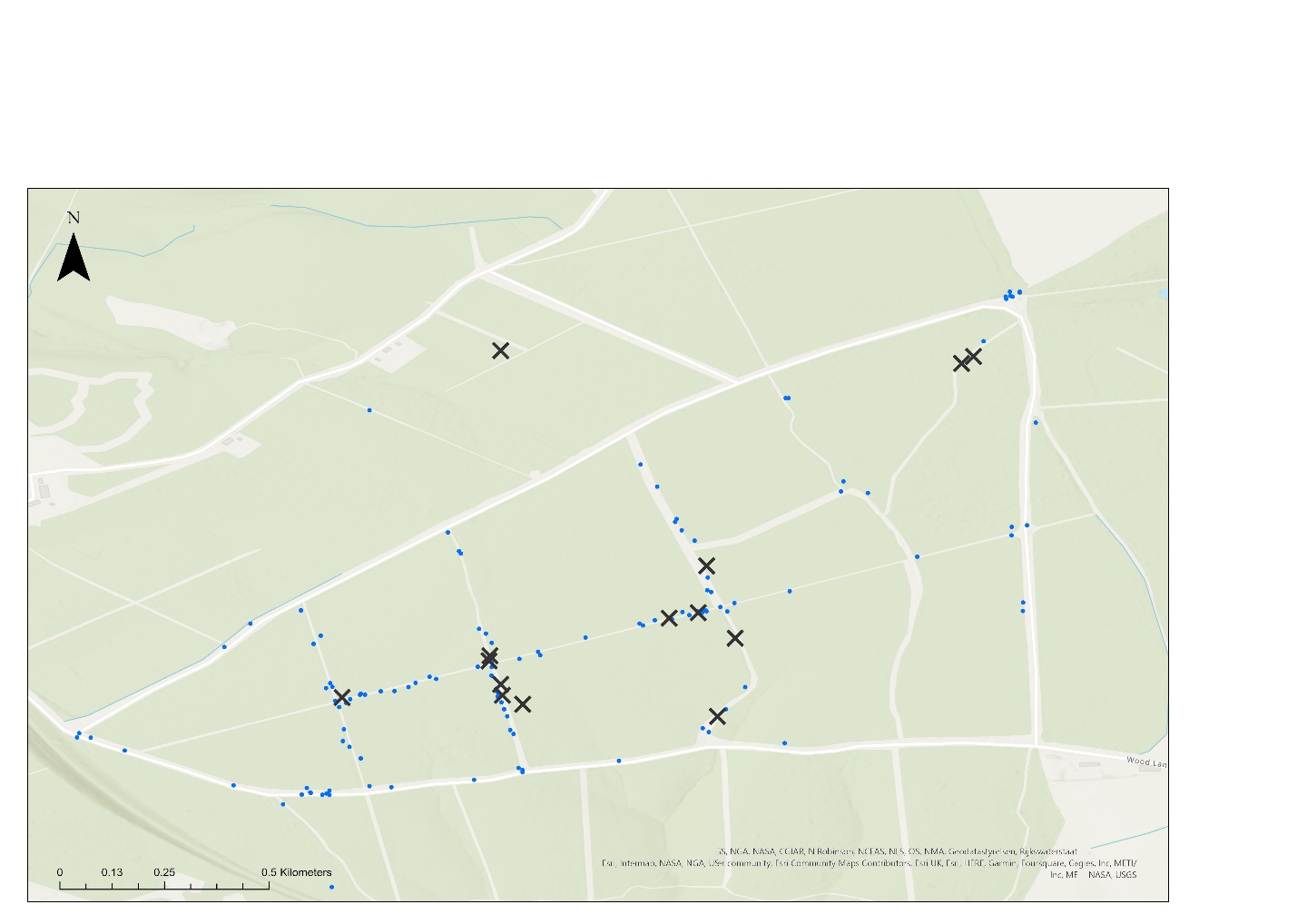


**Supplementary Figure 1: Map of Fineshade.** Map of the reintroduction site and wing-clip sample locations shown as black crosses. Blue dots indicate survey data of adult *C. palaemon* in 2022.

**Supplementary Figure 2: Genomic library fragment size distribution.** Agilent Bioanalyzer trace of the pooled genomic library made from 121 individual wing-clips, collected from *Carterocephalus palaemon*.


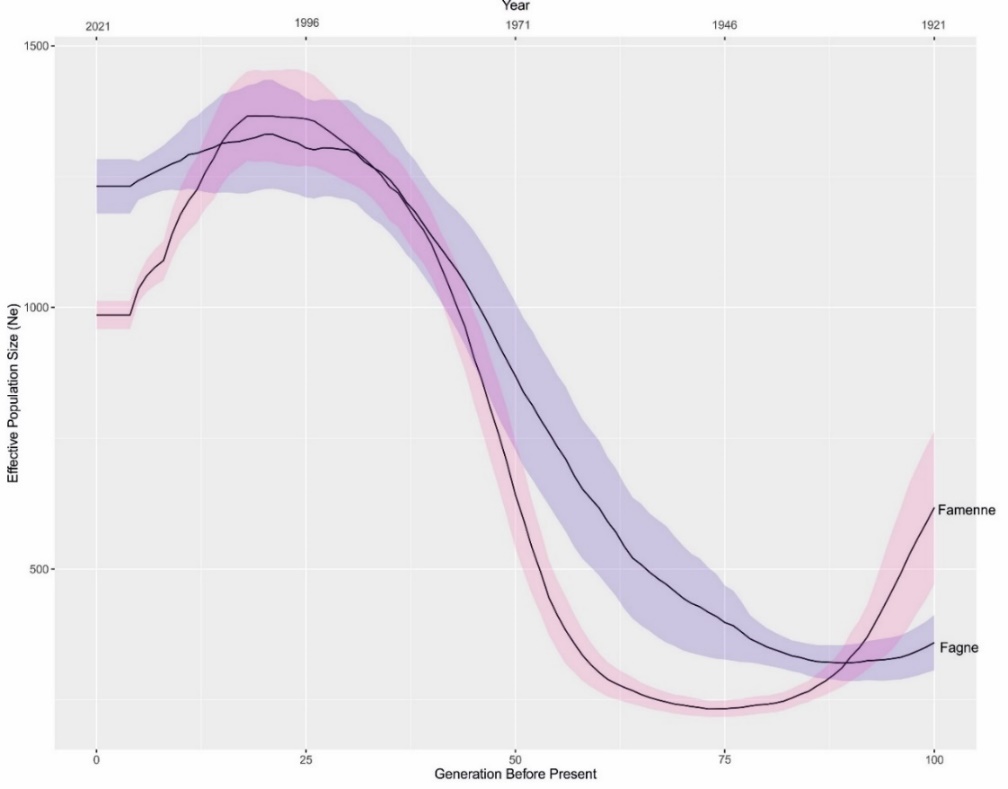


**Supplementary Figure 3. Reconstructed profile of the effective size of the populations in the Fagne and Famenne landscapes in Belgium.** Inferred trajectory of the effective population size (*N*_e_) from the sampled generation (0 = 2021) to 100 years previously, estimated with GoNe.


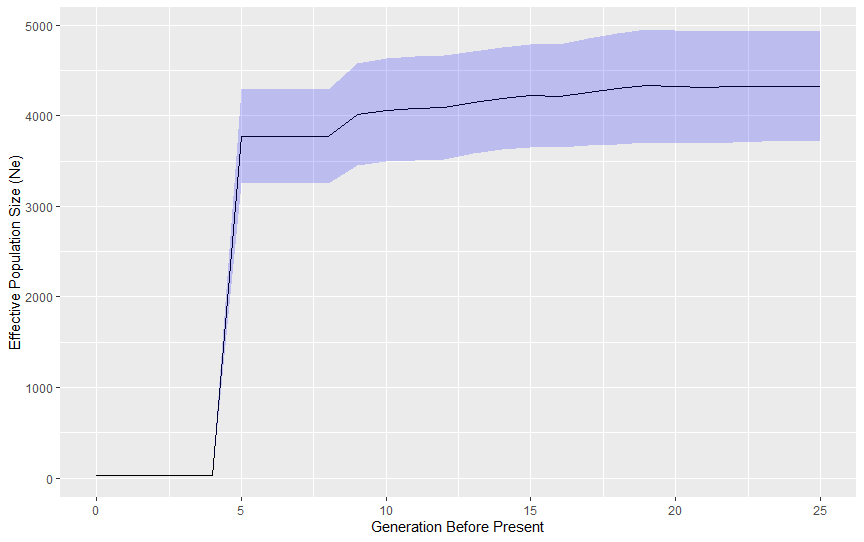


**Supplementary Figure 4. Reconstructed profile of the effective size of the reintroduced population in Fineshade.** Inferred trajectory of the effective population size (*N*_e_) from the sampled generation (0 = 2021) to 25 years previously, estimated with GoNe. *N*_e_ post reintroduction (0-4 years before present) is estimated at 32.7 individuals.

# Supplementary text: Appendix A. Inference of dispersal distance

One important aim of our study was to estimate the average per-generation dispersal of *C. palaemon*, to better inform future conservation of the species. However, this analysis was more uncertain than we had expected due to the distinct genetic clustering of the Fagne and Famenne clades. Though the distances between sites within each landscape are not trivial (0.5-14 km; Suppl. Figure 5), the sample sizes for this analysis are small (5 sites each), and therefore the analysis power is low. Based on the ecology of this species, and other similar butterflies, it is highly likely that most dispersal occurs within-landscapes, and therefore it is still meaningful to report the results of this analysis (Suppl. Table 1, columns 1-6). A typical dispersal distance of c. 1 km within a complex of connected habitat is perhaps higher than expected for a small butterfly, but is not outside of previous estimated dispersal ranges for this species (Ravenscroft, 1994) and may help to explain how this species persists at a low population density (Ravenscroft, 1994; Wildman, 2023).

By contrast, when we include all sampling sites in the dispersal analysis (all the points in Suppl. Figure 5), the power is higher, but the assumptions of the analysis are more likely to be violated. If any factor *in addition to distance* is causing a lack of genetic exchange between the Fagne and Famenne landscapes, this analysis cannot disentangle them. Since we suspect there may be incompatibility between the two clades (see Discussion section 4.3), this makes us less confident about the dispersal estimate of 2.6-4.6 km (Suppl. Table 1, column 7). Nevertheless, it would not be surprising in general to find a higher dispersal estimate when collecting data over a wider area, if the dispersal kernel is in reality ‘fat-tailed’. The observation that dispersal distances are longer for a small subset of individuals that cross more hostile habitat is common across many animals (Petrovskii and Morozov, 2009), and can have interesting consequences for meta-population dynamics and population genetics (Bohrer, Nathan and Volis, 2005; Petrovskii, Morozov and Li, 2008).

The interesting conclusion we can draw despite the above-mentioned uncertainties is that any dispersal between the Fagne and Famenne populations we have sampled is likely to take several generations to achieve. If we use the strong assumption that the system is in migration-drift equilibrium (Whitlock and McCauley, 1999), the inter-landscape *F*_ST_ of 0.043 implies that 6 individuals are exchanged per generation. This is approximately 0.5% of the population if *N*_e_ is approximately 1000; Suppl. Figure 3. For a Laplacian dispersal kernel, 0.5% of the population travel further than approximately 5 times the mean distance. Therefore, if the kernel mean was 1.2 km, 0.5% of the population would travel further than 6.4 km, and if the kernel mean is at 4.5km, 0.5% of the population would travel over 24 km. In both cases, these distances are much less than the closest of our Fagne sites to Famenne sites (c. 50 km).


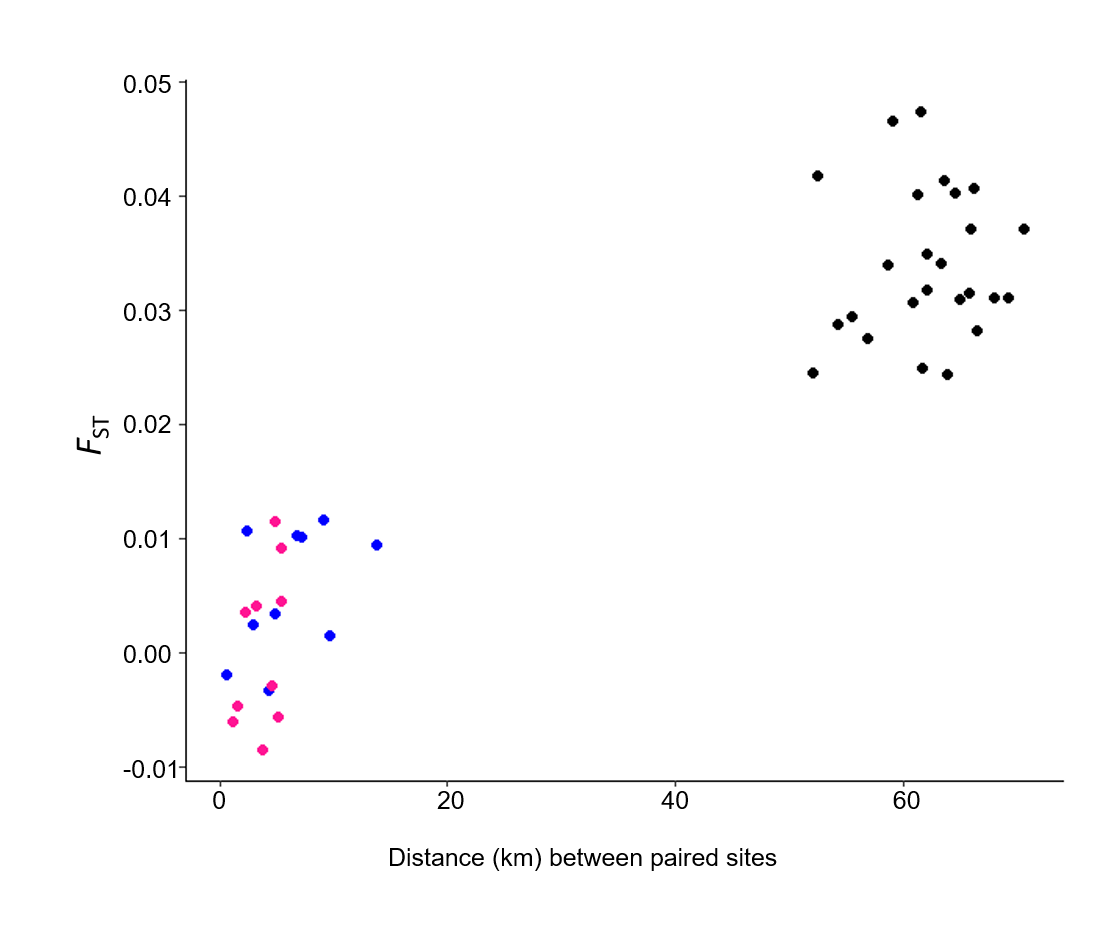


**Supplementary Figure 5. Pairwise *F*_ST_ and geographic distance among Belgian subpopulations of *C. palaemon* in the source region.** *F*_ST_ between sites within the same landscape are shown in blue (Fagne) and pink (Famenne). Black dots represent *F*_ST_ between sites from opposing landscapes.

**Supplementary Table 1. *C. palaemon* dispersal kernel spread and mean dispersal distances in kilometres - based on two assumed dispersal kernel shapes - estimated using data from the Fagne or Famenne landscapes, or all Belgian subpopulations combined**, with their 2.5 and 97.5% confidence limits, incorporating uncertainty both in the regression slope and in Ne. The p-values for the regression slopes were 0.018 for Fagne, 0.202 for Famenne, and <0.001 for both landscapes.

|  | **Fagne** | | | **Famenne** | | | **Both Belgian landscapes** | | |
| --- | --- | --- | --- | --- | --- | --- | --- | --- | --- |
| **Kernel type** | Estimate  (km) | 2.5% | 97.5% | Estimate  (km) | 2.5% | 97.5% | Estimate  (km) | 2.5% | 97.5% |
| Kernel spread | 1.687 | 1.277 | 3.023 | 1.364 | 0.881 | 2.404 | 3.665 | 3.410 | 3.964 |
| Gaussian | 1.119 | 0.902 | 2.137 | 0.965 | 0.623 | 1.700 | 2.591 | 2.411 | 2.802 |
| Laplacian | 1.346 | 1.018 | 2.412 | 1.089 | 0.703 | 1.917 | 4.593 | 4.275 | 4.968 |

**
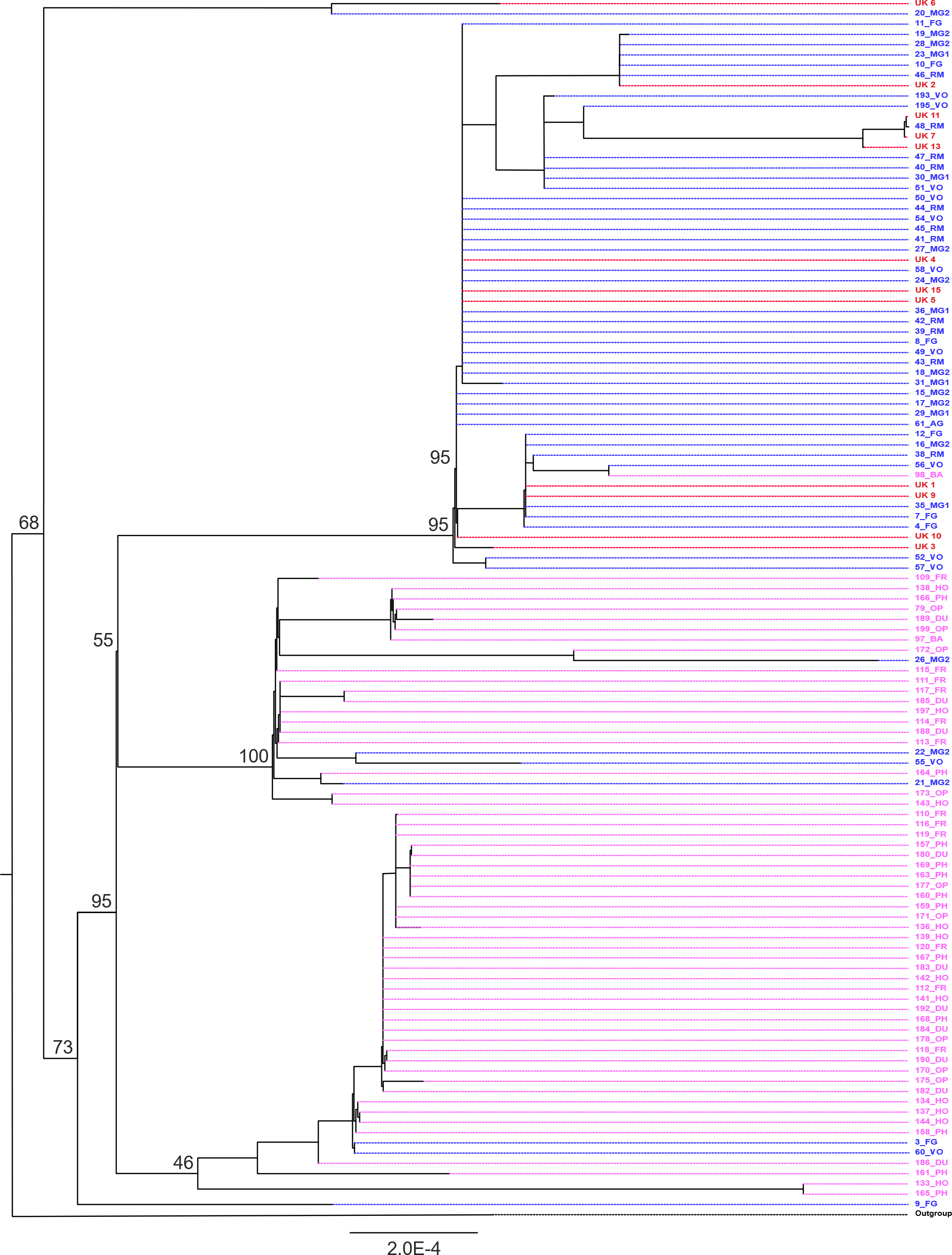
Supplementary Figure 6. Phylogenetic tree of Belgian (source) and Fineshade (reintroduced) *C. palaemon* complete mitochondrial genomes.**  The maximum likelihood tree, with bootstrap support numbers given for the main clades only. Individual labels are shown on each branch end, coloured by the geographic origin landscape: Fagne (blue), Famenne (pink), or Fineshade (red). The reference genome individual from Scotland was used as the technical outgroup.


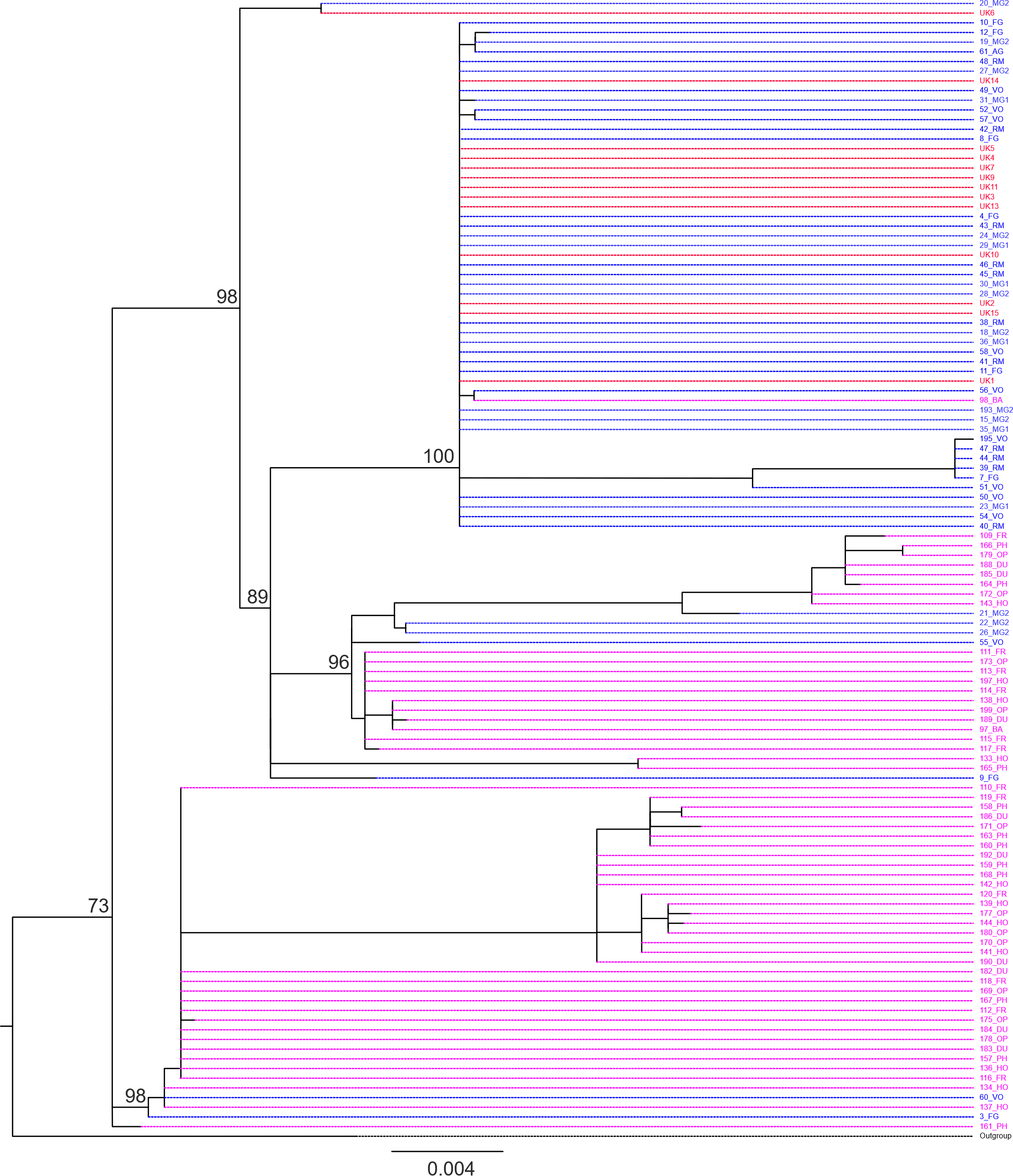
 **Supplementary Figure 7. Phylogenetic tree of *Wolbachia* detected in *C. palaemon* from Belgian (source) and Fineshade (reintroduced) population samples.** The maximum likelihood tree, with bootstrap support numbers given for the main clades only. Individual labels are shown on each branch end, coloured by the geographic origin landscape: Fagne (blue), Famenne (pink), or Fineshade (red). The *Wolbachia* from the reference genome individual from Scotland was used as the technical outgroup.

**Supplementary Table 2**: Table showing how each genotyped *C. palaemon* individual is grouped according to each analysis type. The column for Landscape gives the place of capture of the individual. In the columns PCA and Admixture, “Fagne” means a Fagne-like genetic type, and “Famenne” Famenne-like, according to the PC1 value or the k=2 analysis, respectively. For the mitochondrial and *Wolbachia* tree analyses, the main branches have been classified as majority-Fagne or majority-Famenne, with peripheral branches not classified (see trees in Supplementary Figures 6 and 7).

| Indiv-idual | Sex | Site | Landscape | PCA | Admix-ture | Mitochondrial tree | Wolbachia Tree |
| --- | --- | --- | --- | --- | --- | --- | --- |
| 3 | Male | Fagnolle | Fagne | Fagne | Fagne | Famenne main branch | Famenne main branch |
| 4 | Male | Fagnolle | Fagne | Fagne | Fagne | Fagne branch | Fagne branch |
| 7 | Male | Fagnolle | Fagne | Fagne | Fagne | Fagne branch | Fagne branch |
| 8 | Male | Fagnolle | Fagne | Fagne | Fagne | Fagne branch | Fagne branch |
| 9 | Male | Fagnolle | Fagne | Fagne | Famenne | Peripheral branch | Peripheral branch |
| 10 | Male | Fagnolle | Fagne | Fagne | Fagne | Fagne branch | Fagne branch |
| 11 | Male | Fagnolle | Fagne | Fagne | Fagne | Fagne branch | Fagne branch |
| 12 | Male | Fagnolle | Fagne | Fagne | Fagne | Fagne branch | Fagne branch |
| 15 | Male | Matagne-la-Grande2 | Fagne | Fagne | Fagne | Fagne branch | Fagne branch |
| 17 | Male | Matagne-la-Grande2 | Fagne | N/A | N/A | Fagne branch | N/A |
| 18 | Male | Matagne-la-Grande2 | Fagne | Fagne | Fagne | Fagne branch | Fagne branch |
| 19 | Male | Matagne-la-Grande2 | Fagne | Fagne | Fagne | Fagne branch | Fagne branch |
| 20 | Male | Matagne-la-Grande2 | Fagne | Fagne | Fagne | Peripheral branch (with UK6) | Peripheral branch (with UK6) |
| 21 | Male | Matagne-la-Grande2 | Fagne | N/A | N/A | Famenne branch 2 | Famenne branch 2 |
| 22 | Male | Matagne-la-Grande2 | Fagne | N/A | N/A | Famenne branch 2 | Famenne branch 2 |
| 23 | Male | Matagne-la-Grande1 | Fagne | Fagne | Fagne | Fagne branch | Fagne branch |
| 24 | Male | Matagne-la-Grande2 | Fagne | Fagne | Fagne | Fagne branch | Fagne branch |
| 26 | Male | Matagne-la-Grande2 | Fagne | Fagne | Fagne | Famenne branch 2 | Famenne branch 2 |
| 27 | Male | Matagne-la-Grande2 | Fagne | Fagne | Fagne | Fagne branch | Fagne branch |
| 28 | Male | Matagne-la-Grande2 | Fagne | Fagne | Fagne | Fagne branch | Fagne branch |
| 29 | Male | Matagne-la-Grande1 | Fagne | Fagne | Fagne | Fagne branch | Fagne branch |
| 30 | Female | Matagne-la-Grande1 | Fagne | Fagne | Fagne | Fagne branch | Fagne branch |
| 31 | Female | Matagne-la-Grande1 | Fagne | Fagne | Fagne | Fagne branch | Fagne branch |
| 35 | Male | Matagne-la-Grande1 | Fagne | Fagne | Fagne | Fagne branch | Fagne branch |
| 36 | Male | Matagne-la-Grande1 | Fagne | Fagne | Fagne | Fagne branch | Fagne branch |
| 38 | Male | Romeree | Fagne | Fagne | Fagne | Fagne branch | Fagne branch |
| 39 | Male | Romeree | Fagne | Fagne | Fagne | Fagne branch | Fagne branch |
| 40 | Male | Romeree | Fagne | Fagne | Fagne | Fagne branch | Fagne branch |
| 41 | Female | Romeree | Fagne | Fagne | Fagne | Fagne branch | Fagne branch |
| 42 | Female | Romeree | Fagne | Fagne | Fagne | Fagne branch | Fagne branch |
| 43 | Female | Romeree | Fagne | Fagne | Fagne | Fagne branch | Fagne branch |
| 44 | Male | Romeree | Fagne | Fagne | Fagne | Fagne branch | Fagne branch |
| 45 | Male | Romeree | Fagne | Fagne | Fagne | Fagne branch | Fagne branch |
| 46 | Male | Romeree | Fagne | Fagne | Fagne | Fagne branch | Fagne branch |
| 47 | Male | Romeree | Fagne | Fagne | Fagne | Fagne branch | Fagne branch |
| 48 | Male | Romeree | Fagne | Fagne | Fagne | Fagne branch | Fagne branch |
| 49 | Female | Vodelee | Fagne | Fagne | Fagne | Fagne branch | Fagne branch |
| 50 | Male | Vodelee | Fagne | Fagne | Fagne | Fagne branch | Fagne branch |
| 51 | Male | Vodelee | Fagne | Fagne | Fagne | Fagne branch | Fagne branch |
| 52 | Male | Vodelee | Fagne | Fagne | Fagne | Fagne branch | Fagne branch |
| 54 | Male | Vodelee | Fagne | Fagne | Fagne | Fagne branch | Fagne branch |
| 55 | Male | Vodelee | Fagne | Fagne | Fagne | Famenne branch 2 | Famenne branch 2 |
| 56 | Male | Vodelee | Fagne | Fagne | Fagne | Fagne branch | Fagne branch |
| 57 | Male | Vodelee | Fagne | Fagne | Fagne | Fagne branch | Fagne branch |
| 58 | Male | Vodelee | Fagne | Fagne | Fagne | Fagne branch | Fagne branch |
| 60 | Male | Vodelee | Fagne | Famenne | Famenne | Famenne main branch | Famenne main branch |
| 61 | Male | Agimont | Fagne | Fagne | Fagne | Fagne branch | Fagne branch |
| 193 | Male | Matagne-la-Grande1 | Fagne | Fagne | Fagne | Fagne branch | Fagne branch |
| 195 | Male | Vodelee | Fagne | Fagne | Fagne | Fagne branch | Fagne branch |
| 97 | Female | Baillonville | Famenne | Famenne | Fagne | Famenne branch 2 | Famenne branch 2 |
| 98 | Male | Baillonville | Famenne | Fagne | Fagne | Fagne branch | Fagne branch |
| 109 | Male | Fronville | Famenne | Famenne | Famenne | Famenne branch 2 | Famenne branch 2 |
| 110 | Male | Fronville | Famenne | Famenne | Famenne | Famenne main branch | Famenne main branch |
| 111 | Female | Fronville | Famenne | Famenne | Famenne | Famenne branch 2 | Famenne branch 2 |
| 112 | Male | Fronville | Famenne | Famenne | Famenne | Famenne main branch | Famenne main branch |
| 113 | Male | Fronville | Famenne | Famenne | Famenne | Famenne branch 2 | Famenne branch 2 |
| 114 | Female | Fronville | Famenne | Famenne | Famenne | Famenne branch 2 | Famenne branch 2 |
| 115 | Female | Fronville | Famenne | N/A | N/A | Famenne branch 2 | Famenne branch 2 |
| 116 | Female | Fronville | Famenne | Famenne | Famenne | Famenne main branch | Famenne main branch |
| 117 | Female | Fronville | Famenne | Famenne | Famenne | Famenne branch 2 | Famenne branch 2 |
| 118 | Female | Fronville | Famenne | Famenne | Famenne | Famenne main branch | Famenne main branch |
| 119 | Female | Fronville | Famenne | Famenne | Famenne | Famenne main branch | Famenne main branch |
| 120 | Male | Fronville | Famenne | Famenne | Famenne | Famenne main branch | Famenne main branch |
| 133 | Female | Hotton | Famenne | Famenne | Famenne | Famenne main branch | Peripheral branch (with 165) |
| 134 | Female | Hotton | Famenne | Famenne | Famenne | Famenne main branch | Famenne main branch |
| 136 | Female | Hotton | Famenne | Famenne | Famenne | Famenne main branch | Famenne main branch |
| 137 | Male | Hotton | Famenne | Famenne | Famenne | Famenne main branch | Famenne main branch |
| 138 | Male | Hotton | Famenne | Famenne | Famenne | Famenne branch 2 | Famenne branch 2 |
| 139 | Male | Hotton | Famenne | Famenne | Famenne | Famenne main branch | Famenne main branch |
| 141 | Female | Hotton | Famenne | Famenne | Famenne | Famenne main branch | Famenne main branch |
| 142 | Female | Hotton | Famenne | Famenne | Famenne | Famenne main branch | Famenne main branch |
| 143 | Female | Hotton | Famenne | Famenne | Famenne | Famenne branch 2 | Famenne branch 2 |
| 144 | Male | Hotton | Famenne | Famenne | Famenne | Famenne main branch | Famenne main branch |
| 157 | Male | PetitHan | Famenne | Famenne | Famenne | Famenne main branch | Famenne main branch |
| 158 | Male | PetitHan | Famenne | Famenne | Famenne | Famenne main branch | Famenne main branch |
| 159 | Male | PetitHan | Famenne | Famenne | Famenne | Famenne main branch | Famenne main branch |
| 160 | Male | PetitHan | Famenne | Famenne | Famenne | Famenne main branch | Famenne main branch |
| 161 | Male | PetitHan | Famenne | Famenne | Famenne | Famenne main branch | Peripheral Branch |
| 163 | Male | PetitHan | Famenne | Famenne | Famenne | Famenne main branch | Famenne main branch |
| 164 | Male | PetitHan | Famenne | Famenne | Famenne | Famenne branch 2 | Famenne branch 2 |
| 165 | Male | PetitHan | Famenne | Famenne | Famenne | Famenne main branch | Peripheral branch (with 133) |
| 166 | Male | PetitHan | Famenne | Famenne | Famenne | Famenne branch 2 | Famenne branch 2 |
| 167 | Male | PetitHan | Famenne | Famenne | Famenne | Famenne main branch | Famenne main branch |
| 168 | Female | PetitHan | Famenne | Famenne | Famenne | Famenne main branch | Famenne main branch |
| 169 | Female | Oppagne | Famenne | Famenne | Famenne | Famenne main branch | Famenne main branch |
| 170 | Male | Oppagne | Famenne | Famenne | Famenne | Famenne main branch | Famenne main branch |
| 171 | Female | Oppagne | Famenne | Famenne | Famenne | Famenne main branch | Famenne main branch |
| 172 | Male | Oppagne | Famenne | Famenne | Famenne | Famenne branch 2 | Famenne branch 2 |
| 173 | Male | Oppagne | Famenne | Famenne | Famenne | Famenne branch 2 | Famenne branch 2 |
| 175 | Female | Oppagne | Famenne | Famenne | Famenne | Famenne main branch | Famenne main branch |
| 177 | Male | Oppagne | Famenne | Famenne | Famenne | Famenne main branch | Famenne main branch |
| 178 | Male | Oppagne | Famenne | Famenne | Famenne | Famenne main branch | Famenne main branch |
| 179 | Male | Oppagne | Famenne | Famenne | Famenne | Famenne branch 2 | Famenne branch 2 |
| 180 | Male | Oppagne | Famenne | Famenne | Famenne | Famenne main branch | Famenne main branch |
| 182 | Male | Durbuy | Famenne | Famenne | Famenne | Famenne main branch | Famenne main branch |
| 183 | Female | Durbuy | Famenne | Famenne | Famenne | Famenne main branch | Famenne main branch |
| 184 | Male | Durbuy | Famenne | Famenne | Famenne | Famenne main branch | Famenne main branch |
| 185 | Male | Durbuy | Famenne | Famenne | Famenne | Famenne branch 2 | Famenne branch 2 |
| 186 | Female | Durbuy | Famenne | Famenne | Famenne | Famenne main branch | Famenne main branch |
| 188 | Female | Durbuy | Famenne | Famenne | Famenne | Famenne branch 2 | Famenne branch 2 |
| 189 | Male | Durbuy | Famenne | Famenne | Famenne | Famenne branch 2 | Famenne branch 2 |
| 190 | Female | Durbuy | Famenne | Famenne | Famenne | Famenne main branch | Famenne main branch |
| 192 | Male | Durbuy | Famenne | Famenne | Famenne | Famenne main branch | Famenne main branch |
| 197 | Female | Hotton | Famenne | Famenne | Famenne | Famenne branch 2 | Famenne branch 2 |
| 199 | Female | Oppagne | Famenne | Famenne | Famenne | Famenne branch 2 | Famenne branch 2 |
| UK1 | Male | Fineshade | FIneshade | Fagne | Fagne | Fagne | Fagne |
| UK2 | Male | Fineshade | FIneshade | Fagne | Fagne | Fagne | Fagne |
| UK3 | Male | Fineshade | FIneshade | Fagne | Fagne | Fagne | Fagne |
| UK4 | Male | Fineshade | FIneshade | Fagne | Fagne | Fagne | Fagne |
| UK5 | Female | Fineshade | FIneshade | Fagne | Fagne | Fagne | Fagne |
| UK6 | Male | Fineshade | FIneshade | Fagne | Fagne | Peripheral branch (with 20) | Peripheral branch (with 20) |
| UK7 | Male | Fineshade | FIneshade | Fagne | Fagne | Fagne | Fagne |
| UK9 | Female | Fineshade | FIneshade | Fagne | Fagne | Fagne | Fagne |
| UK10 | Male | Fineshade | FIneshade | Fagne | Fagne | Fagne | Fagne |
| UK11 | Male | Fineshade | FIneshade | Fagne | Fagne | Fagne | Fagne |
| UK13 | Female | Fineshade | FIneshade | Fagne | Fagne | Fagne | Fagne |
| UK14 | Female | Fineshade | FIneshade | Fagne | Fagne | N/A | Fagne |
| UK15 | Female | Fineshade | FIneshade | Fagne | Fagne | Fagne | Fagne |
